# Biomimetic engineering for water harvesting: 3D printed solutions for arid regions

**DOI:** 10.1039/d5lf00222b

**Published:** 2026-03-04

**Authors:** Henry Apsey, Donald Hill, Shirin Alexander

**Affiliations:** a School of Engineering and Applied Sciences, Department of Chemical Engineering, Swansea University Swansea Wales UK s.alexander@swansea.ac.uk

## Abstract

Approximately one third of the Earth's surface has a climate that is currently considered arid, which in 2020 affected around 2.4 billion people. This extreme condition leads to challenges accessing water for agriculture, hygiene, and sanitation, amongst a myriad of others. Over millions of years, nature has evolved ingenious strategies for survival in such environments. One notable example is the Namib desert tenebrionid beetle, which collects water from fog using its elytra—a shell featuring a pattern of hydrophilic and hydrophobic surfaces. Inspired by this natural mechanism, we have studied 3D-printed green superhydrophilic/superhydrophobic hybrid surfaces and evaluated their water collection performance in a controlled climate chamber, which was designed to simulate arid conditions. The coated surfaces demonstrated a 4–5 fold improvement in water collection efficiency compared to uncoated samples. Both spiked and beetle-inspired designs were tested, revealing that larger spikes or bumps collected nearly twice as much water. Beetle-like channel structures also proved to be highly effective designs. Overall, it was observed that hybrid coatings outperformed fully hydrophilic or hydrophobic surfaces, suggesting that the increased complexity does indeed lead to greater water harvesting efficacy. These findings offer valuable insights into the design and engineering of efficient and sustainable water harvesting technologies for arid regions.

## Abstract 2

A biomimetic 3D-printed surface coating inspired by the Namib desert beetle, dune Bushman grass, and rice leaves enables efficient fog harvesting through spatially patterned hydrophilic–hydrophobic regions.

## Graphical abstract

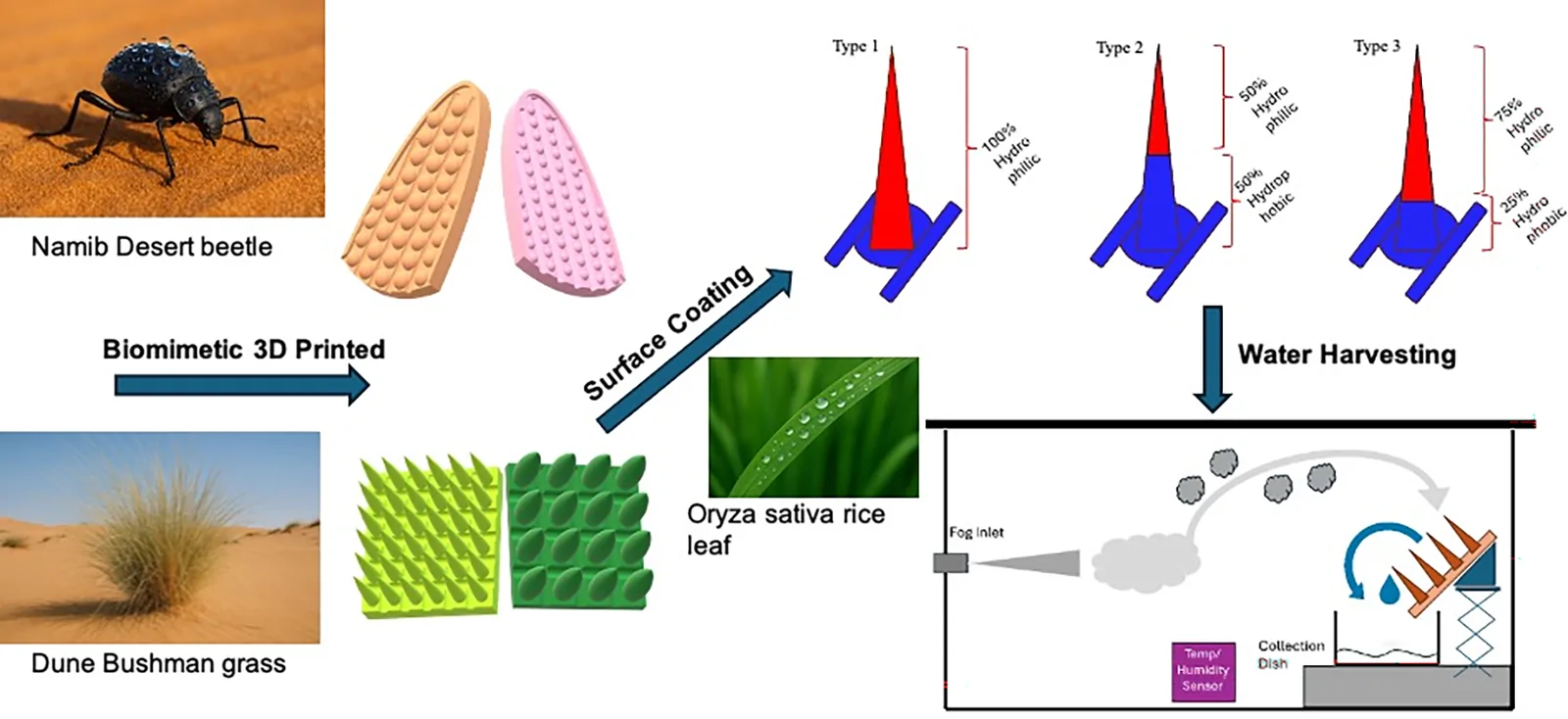

## 1. Introduction

Global water shortages pose significant risks to both human populations and ecosystems, with water scarcity creating risks to agricultural productivity, human health, ecosystem degradation and having the potential to lead to conflict and social unrest.^1^ In line with the UN Sustainable Development Goal 6 for clean water and sanitation, fog collection has been attracting attention due to its potential to address water scarcity in regions where conventional water sources are limited.^2–7^ Fog collection is particularly well-suited for arid and semi-arid regions, particularly in locations where fog is a regular and predictable meteorological phenomenon. As these regions often experience water scarcity, innovative solutions may be able to augment existing water supplies. Some countries, such as Chile, Peru, and California, have successfully implemented this method in their coastal areas, as well as mountainous regions in countries like Morocco and Nepal,^8–13^ providing a sustainable and environmentally friendly alternative to traditional water sources in regions where fog is abundant but rainfall is scarce. However, the efficiency of fog collection can vary depending on factors such as fog density, wind patterns, and the efficiency of the collection system. Ongoing research and technological advancements continue to improve the effectiveness and scalability of fog collection systems.^2,4–6,11^

For next-generation technologies, natural biological sources can provide valuable insights and solutions for improving current and future water collection systems, making them more efficient, sustainable, and adaptable to a changing world. Over millions of years of evolution, nature has evolved topographic structures that are highly effective for controlling the flow of water for nutrient collection or so that it can be better directed to where it is needed. A key example of this is *Oryza sativa*, the rice plant, which shows anisotropic wetting to help deliver water droplets towards the plant's roots. This wettability arises due to the series of microstructural ridges present on the leaf's surface that are coated with nanoscale wax structures (Fig. S2). This microstructural feature causes the surface to show Cassie–Baxter wetting only in the parallel direction, which directs the shedding of water droplets to the roots. By comparison, droplet flow perpendicular to this requires a greater force to overcome the topographic effect caused by the ridges, which results far higher sliding angles. Directing the flow of water to the soil directly beneath it facilitates the flow of nutrients and helps the plant to maintain its turgor pressure.^14^ These naturally optimised wetting gradients highlight how rice leaves balance water management with biological performance, offering a compelling model for nature-inspired engineering.^15,16^ Another key impressive example where evolution has led to the creation of impressive water-harvesting structures is in the case of the Namibian desert beetle (*Stenocara gracilipes*). The beetle has a unique shell structure with hydrophilic bumps on its back. These bumps are surrounded by hydrophobic troughs that are coated with a waxy substance to repel water. This combination of hydrophilic and hydrophobic surfaces creates a surface tension gradient that causes water droplets to condense from the fog onto the beetle's back. In the early morning, when fog blankets the desert, the beetle climbs to the top of the dunes and positions itself facing into the wind. As fog passes over the beetle, water droplets in the fog adhere to its hydrophilic bumps. These water droplets gradually accumulate on the beetle's back, forming larger droplets, and as the droplets reach a critical size, they roll down the hydrophobic surface and collect in the beetle's mouth, providing scarce water in an otherwise arid environment.^17–19^ In the same geographical region of Southern Africa, the Dune Bushman grass (*Stipagrostis sabulicola*), a plant native to the Namib desert, also utilises a similar approach for water collection. Similar to the beetle, the hydrophilic hairs on the its leaves provide condensation sites for water droplet present in the fog.^20^ Its unique long, narrow leaf structure rolled into a tight cylinder helps to minimise evaporative water losses from transpiration. As the fog passes over the grass, the droplets adhere to the hydrophilic areas, and are then channelled to the plant's roots through regions coated with a waxy hydrophobic substance. This allows the Dune Bushman grass to survive in the arid conditions of the Namib desert, where rainfall is extremely scarce.^20,21^ By comparison, humans have utilised large nets in fog collection systems, which are typically made of fine meshes of polyethylene or polypropylene. This mesh is designed to have small openings that allow fog droplets to pass through but capture droplets as they collide with the net fibres before they flow down into collection vessels.^22,23^

At the lab-scale, surfaces can be laser or chemically etched to create microscale or nanoscale features that enhance water droplet nucleation and capture. These surface modifications can also include microtextures, hydrophilic coatings, or other nanostructures.^24^ Electrospun membranes can also be designed with specific properties to enhance fog collection efficiency by providing a high surface area for water droplet capture.^25^ 3D printing, a relatively novel approach that involves the use of specific geometries, is also optimised for fog collection. The structures it can create can be designed with intricate patterns to maximise water droplet capture efficiency. The technique is relatively low-cost and allows scalable high-speed prototyping to take place to create three-dimensional objects by adding material layer by layer of printing material based on a digital design.^26–29^ Fused deposition modelling (FDM) is achieved by taking a solid thermoplastic filament (commonly polylactic acid (PLA)) and then channelling the filament through a heated extrusion head, where it is melted and then deposited through a nozzle where it solidifies into the desired design. The design being printed is subsequently lowered allowing its height to grow in the vertical direction.^30^ As FDM is a layered technique that unavoidably provides a roughness to the surface, this impacts the wettability by promoting droplet pinning.^31^ Designing a system that collects both the water droplets from the design but also factors in water runoff requires consideration of channel type, depth, and direction.

Previously our group has studied the mechanical and environmental durability, including durability, abrasion resistance, and adhesion of nanoparticle coatings.^32–34^ These works report robust performance under standardised tests; the results of which are directly relevant here because of the ease at which the particles can be sprayed onto surfaces with complex geometries. Changing the areal ratio of hydrophobic to hydrophilic regions on a surface can impact water collection and mobility. For example, increasing the proportion of hydrophilic zones on a surface enhances the surface's affinity for water molecules, promoting the adsorption and retention of liquid water. Contrastingly, a higher ratio of hydrophobic regions reduces the surface area available for droplet pinning and can promote shedding or runoff.^35,36^

This research has used inspiration from the natural structures found on the Namib beetle, rice leaf, and dune bushman grass to design 3D-printed sustainable superhydrophilic/superhydrophobic hybrid surfaces and to evaluate their water collection performance at the laboratory scale using a climate chamber to mimic foggy environments. Designs with features of a range of shapes and sizes were evaluated in addition to a hydrophobic, hydrophilic and hybrid coating comparison. All of these variables were found to influence the quantity of water recovered and could thus provide insight into the future design of effective and environmentally friendly water harvesting devices.

## 2. Materials and methods

### 2.1. Initial designs and printing

Designs were produced in Solidworks, Autodesk Fusion 360 and Autodesk Tinkercad. These were then sliced using Ultimaker Cura (with the plugin MeshTools) and Creality Slicer at the default setting for the printers unless stated otherwise. Designs were printed using Ultimaker 3 and Creality Ender 3 V2 printers using Steadytech 1.75 mm PLA filament. Printing accuracies are as follows: *X*–*Y* dimensional accuracy ±0.03–0.05 mm, *Z* accuracy ±0.02–0.03 mm, layer height precision ±0.01 mm (typical layer heights 0.08–0.16 mm), and minimum reliable feature size ∼0.2–0.3 mm. Note that overall dimensional error is dominated by material shrinkage rather than tool precision. Resin designs were sliced with FormLabs Preform and printed on a Formlabs Form 3+. The surface area of each design was calculated using the software “Meshy”.^37^

### 2.2. Design of testing methodology for fog collection measurements

**Fig. 1:**
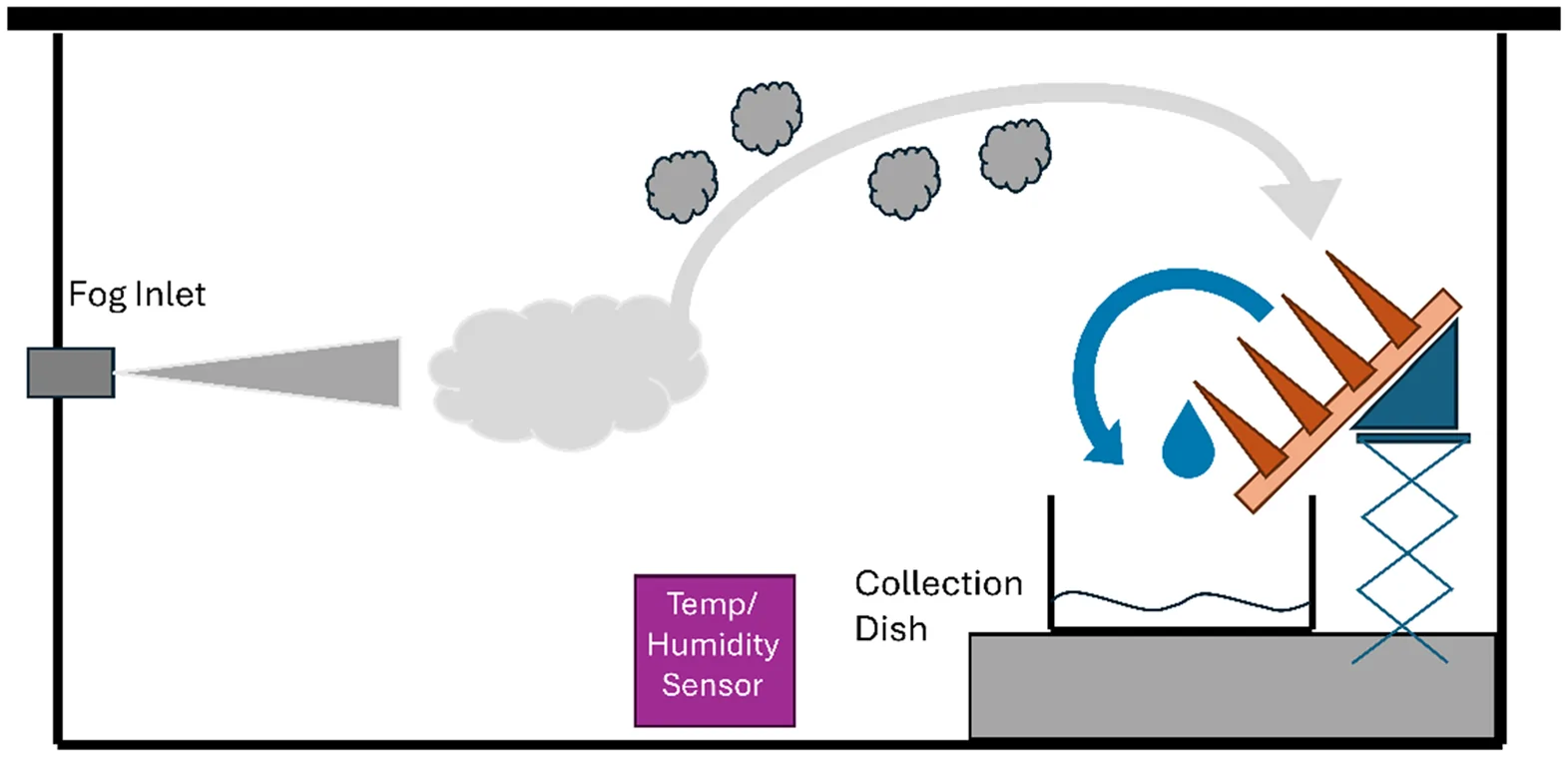
A schematic of the climate chamber used for the water collection experiment.

A biodome (closed environment with fixed ecological characteristics) was set up in a clear acrylic box of 50 × 30 × 25 cm^3^ consisting of a fog inlet from a Swell fog maker, with a maximum 250 ml per hour fog output of deionised “fog” water. Output volume was found to be consistent at a controlled temperature and humidity (20 ± 1°, 52 ± 5%). The input tank of 500 ml was weighed before and after each run. This was controlled by a timer plug, to keep the experiments the same length at 4 hours. At the other end of the box is a height-adjustable tilting block to allow any sample to be attached using double-sided crafting tape. This was fixed at 45° throughout this study based on preliminary rig trials to balance capture and downward transport without introducing shear induced redirection from the baseplate. We observed that much steeper angles deflected incoming fog flow and shallower angles reduced droplet mobility over typical run times. Underneath this was a glass Petri dish to collect the moisture runoff from the samples, with each sample being left for 30 minutes after the fogging had finished, before removal to allow the droplets to run off the samples. The recovery percentage was calculated from the water collected over the water fogged in the tank. Humidity and temperature were measured in the box at the start of each experiment and were thoroughly dried between runs. Airflow was kept consistent throughout the experiment. Control tests were performed with just the collection dish in the box without any printed structures (design e), and then with uncoated structures (designs a–d, i–j and l–o), and structures without spikes/bumps (designs f–h and k). The schematic of the experiment is shown in Fig. 1, and the picture of the climate chamber is shown in Fig. S5. An attempt was made to follow ISO 6270-2-2018, where possible, to allow for repeatability and the determination of resistance to humidity with consideration to condensation.

### 2.3. Materials and synthesis of hydrophobic/hydrophilic nanoparticles

Al_2_O_3_ nanoparticles (NPs) (100 mg, 13 nm mean particle size, average 100 m^2^ g^−1^ specific surface area), 2-2-2-methoxyethoxyethoxy, ethanol, isopropanol, and toluene were purchased from Sigma-Aldrich and used as received. Isostearic acid was purchased from TCI chemicals. Distilled water (Millipore, 18 MΩ cm) was used throughout the experimental process.

Superhydrophobic and superhydrophilic Al_2_O_3_ NPs were synthesised as in previous experimentation.^33,34,38^ Aluminium oxide nanoparticles were refluxed overnight in toluene (100 mL) with the appropriate carboxylic acid: isostearic acid, and 2-[2-(2-methoxyethoxy)ethoxy]acetic acid. The functionalized particles were then centrifuged for 30 minutes and then re-dispersed in 2-propanol (2 × 30 mL) and ethanol (1 × 30 mL) and then centrifuged again to remove unreacted carboxylic acid. Finally, particles were oven-dried at 80 °C overnight. Designs were sprayed with a minimum of 3 layers using a compressed air propellant spray gun with a pressure of 20 psi. Before any testing, models were validated on a contact angle machine where a droplet was placed onto the surface after drying to confirm the coating was effective. Table S1 shows the contact angle of uncoated *vs.* coated plastic types and shows that hydrophilic Al_2_O_3_ particles are able to coat this material and universally make droplets spread out to a thin film with water contact angles of <1°. On samples coated with hydrophobic Al_2_O_3_, all contact angles were >150°, which indicates superhydrophobic performance of the coating with droplets beading up (Fig. S4 shows typical droplet formation). All datasets were tested with a minimum of *n* ≥ 3 replicates.

### 2.4. Mixed hydrophobic/hydrophilic coatings

**Fig. 2:**
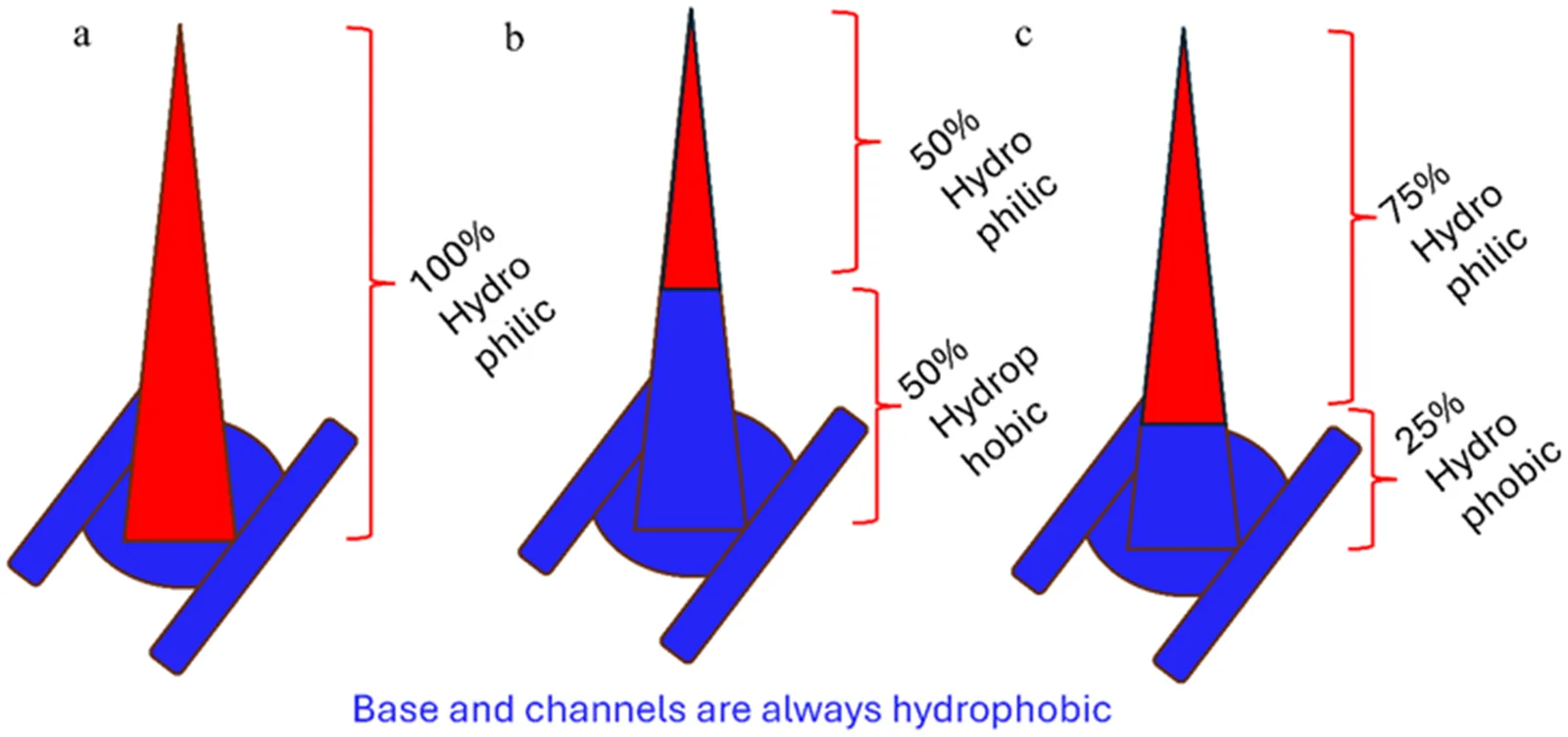
A graphic illustration of the model design of coated spike sections for mixed hydrophilic/hydrophobic coatings of a) type 1: 100/0%, b) type 2: 50/50%, and c) type 3: 75/25%.

Spikes that were designed to recreate the Namib grass were coated fully hydrophilic and the base coated hydrophobic, as is shown in Fig. 2a (type 1). Tests were additionally performed with the coatings either 50/50% (50% of the spike hydrophilic, type 2), and 75/25% (75% of the spike hydrophilic and the rest hydrophobic, type 3) as seen in Fig. 2b and c, respectively. Masking was attempted with laser-cut acrylic, matching the shapes designed, but it was found that the overspill from the spray did not lead to precise, sharp masking lines. Therefore, the top areas (spikes) were masked with liquid latex and masking tape, then the bottom was sprayed with hydrophobic, followed by removing the masking, re-masked with masking tape, the bottom of the design and then the top sections sprayed with hydrophilic particles. Optical images and videos (Fig. S1) are provided in the SI, illustrating the masking procedure and showing precise, sharp masking lines with clearly defined boundaries between the hydrophobic and hydrophilic coatings. Early designs (a–e) were tested using contact angle measurements to confirm that areas were both superhydrophobic or hydrophilic before use. Each sample was robustly tested and underwent a minimum of 3 full fog collection cycles without observable degradation in coating integrity or water collection performance. While long-term outdoor durability is beyond the scope of this study, the coatings were stable across all repeated laboratory experiments presented.

## 3. Results and discussion

### 3.1. Test materials and variations

Some experimentation was performed initially to compare the optimum printing conditions, including speed, temperature, diameter of nozzle, plastic type (polyethylene terephthalate glycol (PETG), polylactic acid (PLA), acrylonitrile butadiene styrene (ABS) and resin). It was decided to use PLA due to cost, availability, and ease of printing with PLA compared to the stronger plastics. Also, PLA is often regarded as a more sustainable alternative to traditional plastics, particularly those derived from fossil fuels like petroleum-based plastics. PLA is made from renewable resources such as corn starch or sugarcane, using feedstocks derived from non-food crops or agricultural waste, making it biodegradable and compostable under the right conditions. Its production typically generates fewer greenhouse gas emissions compared to conventional plastics. Experimentation was also performed on the infill (the density and structure of plastic inside the 3D printed shapes) and the resolution of print to optimise part weight, strength, and printing time. It was decided to opt for a high infill ratio to improve structural integrity, decrease the likelihood of water entering the prints and at a medium resolution to allow for some roughness to the surface, which may aid wettability properties. Although extensively varied in prototyping, the angle of tilt was also kept consistent across the experimentation, but it can lead to significant variation, as, depending on the angle, the fog deflects off the flat surface of the sample into other areas of the test apparatus. It is believed that the fog follows isentropic nozzle flow indirectly through air currents as it moves through the narrow opening of the hose as the fog is introduced into a stream of air flowing through an isentropic nozzle, the expansion of the air as it exits the nozzle could cause a decrease in localised pressure and temperature, potentially leading to condensation or changes in the size and distribution of the fog droplets. This was factored into the design with a large enough distance between the fogging nozzle and the samples to attempt to make this atmospheric rather than localised fogging.^39^

### 3.2. Material durability

In our controlled climatic chamber experiments, no degradation of PLA or coating leaching was observed over the test durations. PLA is generally stable under ambient conditions but can undergo photodegradation (chain scission) and embrittlement under prolonged UV exposure and thermal cycling. UV stabilisers/pigments may extend service life. Long term outdoor aging is identified as an important avenue for future work.^40,41^

### 3.3. Nature-inspired designs

Design patterns are based on three categories: the first one is simple initial designs, the second is based on the desert grass-inspired patterns, and the third is based on the desert beetle-inspired patterns. The details of each design are discussed below. As discussed in the Introduction, the rationale for choosing these designs stemmed from these surfaces' areal or directional effects on the wettability. The surface of the rice leaf also falls into the same class, whereby its microscale channels facilitate droplet pinning perpendicular to channel direction. Ultra-low droplet adhesion, brought about by Cassie–Baxter wetting, is facilitated by the periodic presence of wax structures (Fig. S2). This anisotropic wettability these surfaces possess provided the rationale for incorporating channels and hybrid hydrophobic/hydrophilic coatings into the grass and beetle-inspired designs (sections 3.3.2 and 3.3.3).

#### 3.3.1. Initial designs

**Fig. 3:**
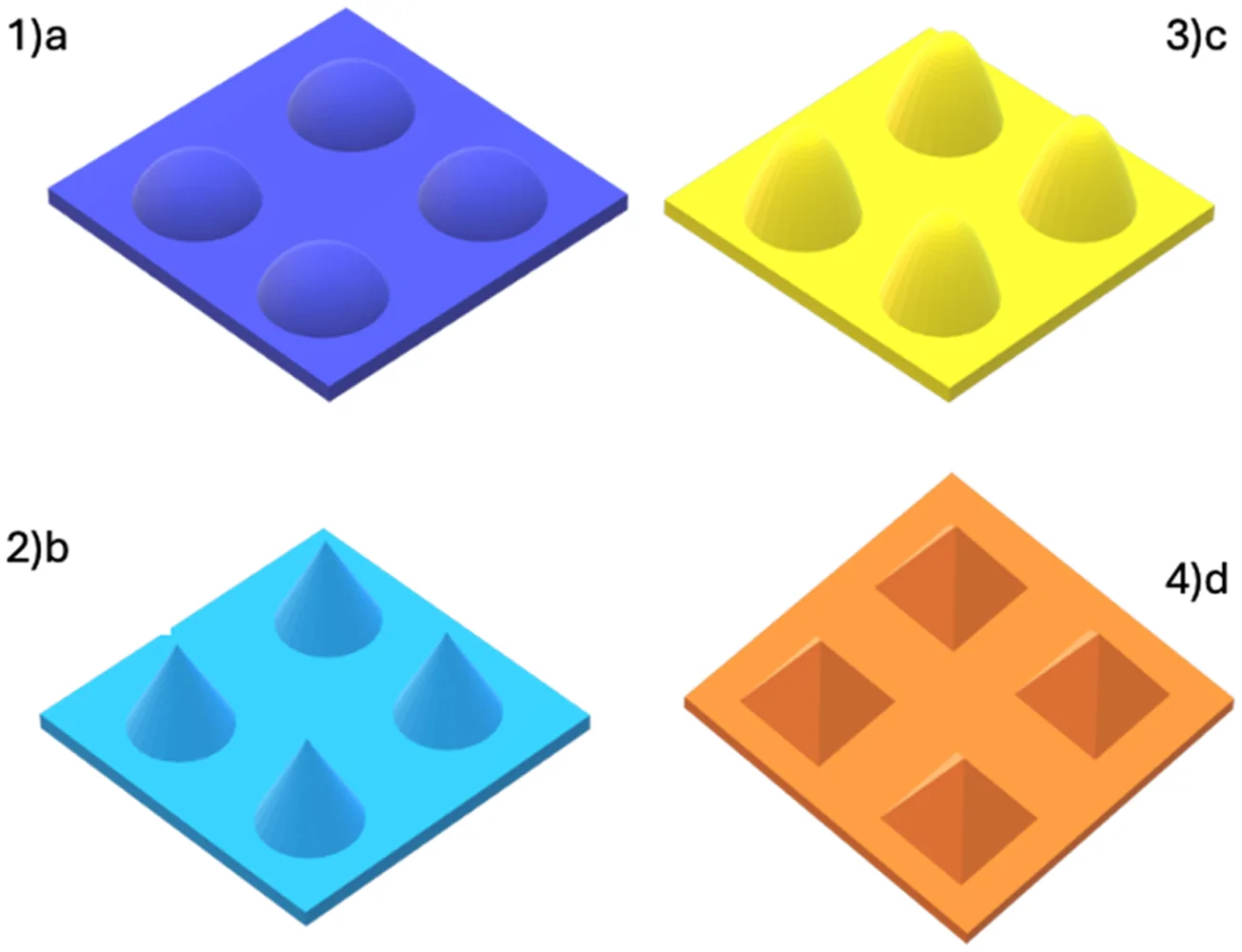
Simple designs of a 4 cm × 4 cm grid of different “bump” shapes for comparison. 1) Design (a) hemispheres, 2) design (b) cones, 3) design (c) paraboloids, and 4) design (d) pyramids.

Initial designs were kept small, with the main points of comparison being the type of shape and the density of “bumps” on a surface. The initial shapes tested were hemisphere (a), cones (b), smooth-topped cones, paraboloid (c), and triangular-based pyramid (d), which are shown in Fig. 3. These were chosen to test a range of 3D shape structures, and although similar, the effect of the vertices, side length can be tested through this comparison. These were tested in 2 × 2 bumps with a 2.5 cm distance between the centres of each bump.

#### 3.3.2. Grass-inspired designs

**Fig. 4:**
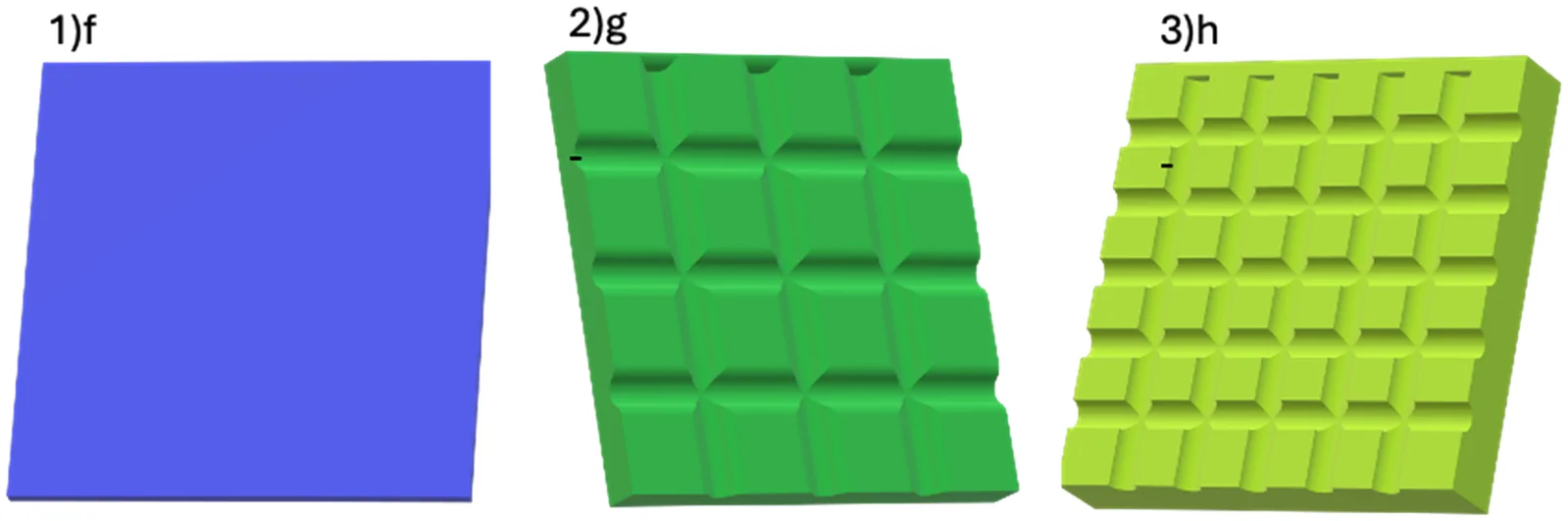
A set of control designs inspired by grass spikes. 1) Design (f), a simple, smooth base used as a control block of PLA, 2) design (g), a control base of the 4 × 4 spike design without the spikes, 3) design (h), a control base of the 6 × 6 spike design without the spikes.

Control tests were performed with base structures of PLA (a printed design of 15 × 15 × 1 cm). Design (f) was a block to look at the effect of a flat surface with no channels. This was compared with designs (g) (channels of 4 × 4) and (h) (channels of 6 × 6) to examine the impact of the channels, and their density without spikes, as shown in Fig. 4. These were chosen as a control design for comparison with the grass-inspired designs.

The next phase of testing featured nature-based designs taking inspiration from the Namib Dune Bushman Grass. These designs had different densities of spikes, along with channel depth and width in the mid-section.

**Fig. 5:**
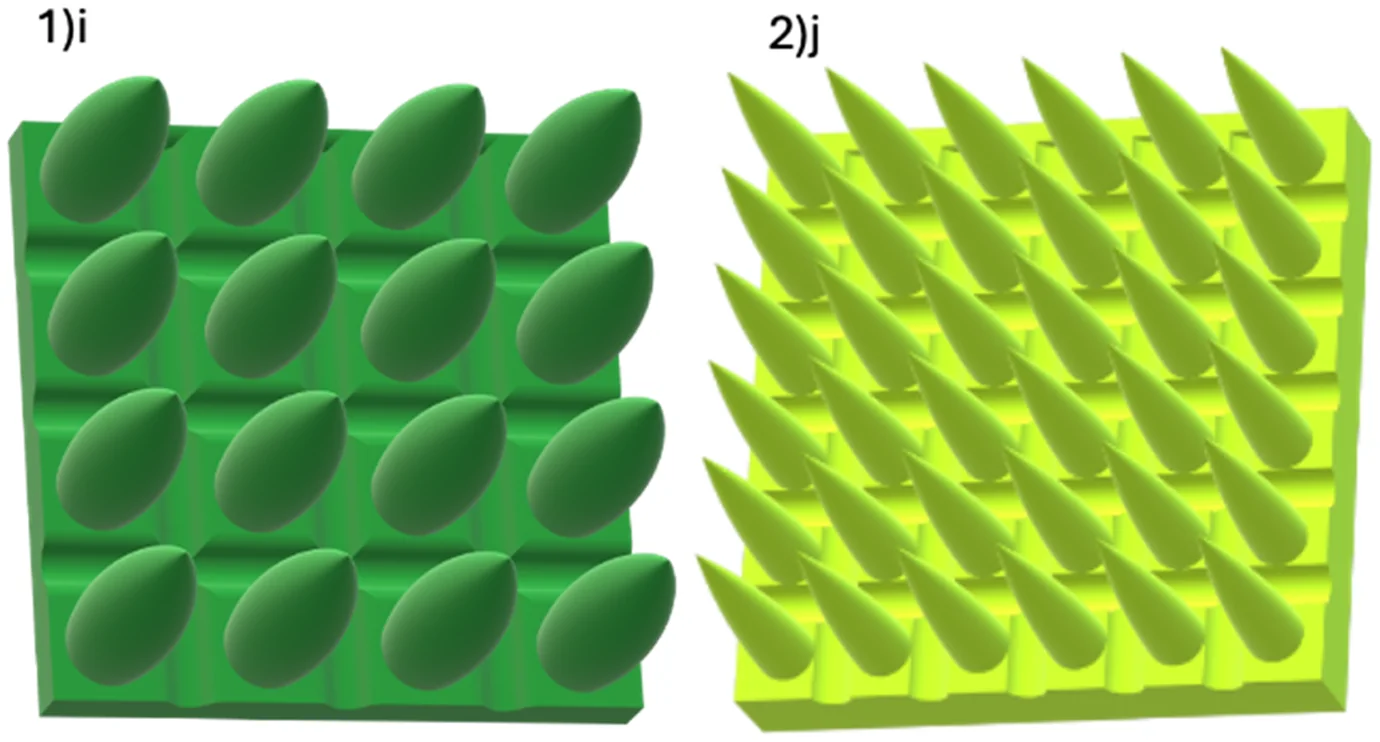
A set of designs inspired by grass spikes. 1) Design (i) a low-density grass-inspired design with 4 spikes across the substrate & 2) design (j) a high-density grass-inspired design with 6 spikes across the substrate.

Initial sketches included the use of a Reuleaux cone, where the distance between two opposite points is the same across the entire model.^42^ This arrangement optimises water capture with a high surface area and then uses gravity to feed this water to the collection base. However, attaching this to the base structure meant the design was too fragile, and therefore, this was lengthened, and the base surface area increased. In a design of 15 × 15 cm, it was chosen to test a 4 × 4 design (i) and a 6 × 6 spikes design (j) of the same height, as shown in Fig. 5.

#### 3.3.3. Beetle-inspired designs

**Fig. 6:**
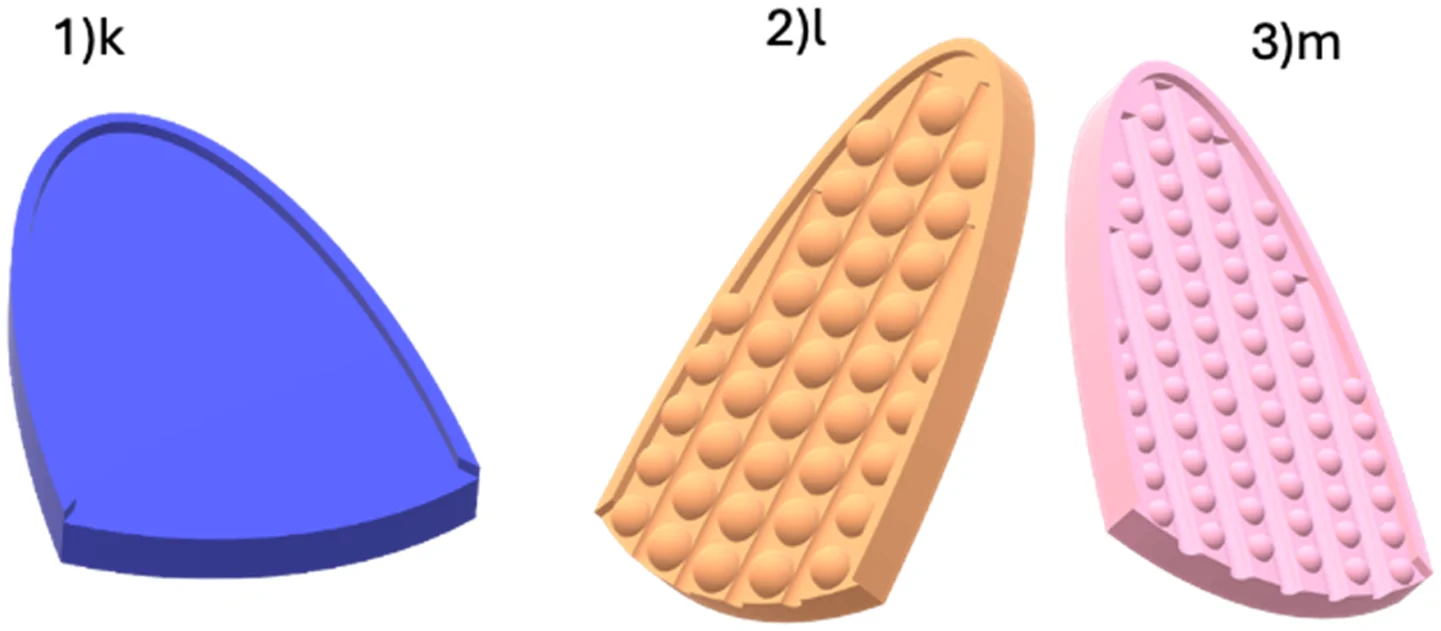
A set of designs inspired by the back of the beetle. 1) Design (k) the basic control, 2) design (l) a flat beetle-inspired design with 5 bumps across the substrate, and 3) design (m) a flat beetle-inspired design with 6 bumps across the substrate.

Taking inspiration from nature, another design was conceived based on the shape of the back of the Namib desert beetle (*Stenocara gracilipes*). Initially, a flat “D” shape base with a raised rim was chosen as a control, design (k). Bumps were added along with wide channels with 5 bumps, design (l) and 6 bumps, design (m), across the substrate to examine the effect of bump size, as shown in Fig. 6.

**Fig. 7:**
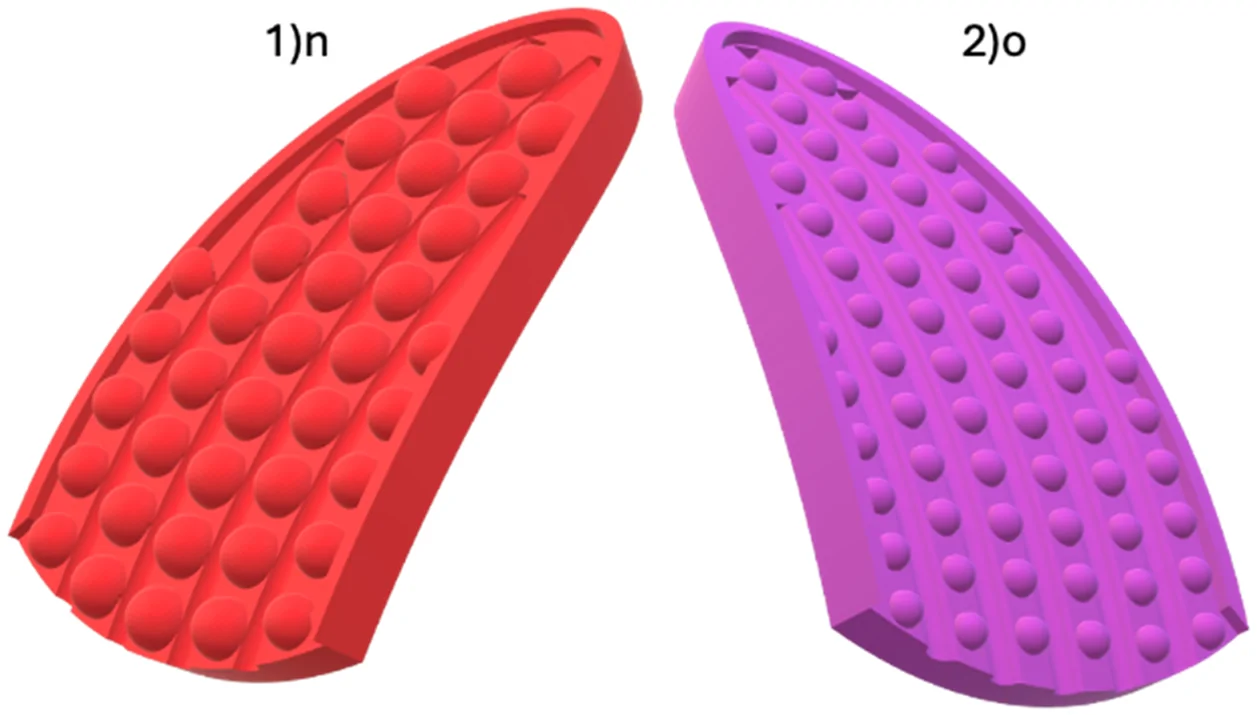
A set of designs inspired by the back of the beetle. 1) Design (n) a bent beetle-inspired design with 5 bumps across the substrate, and 2) design (o) a bent beetle-inspired design with 6 bumps across the substrate.

In its natural habitat, the beetle can elevate its lower back by 23 degrees. This angle allows fog drops to strike the surface and collect dew or fog water by gravity.^43^ In a nature-inspired design, the previous designs were bent by ∼20° to assess the effect of bend *vs.* flat beetle-inspired design, as shown in Fig. 7.

**Fig. 8:**
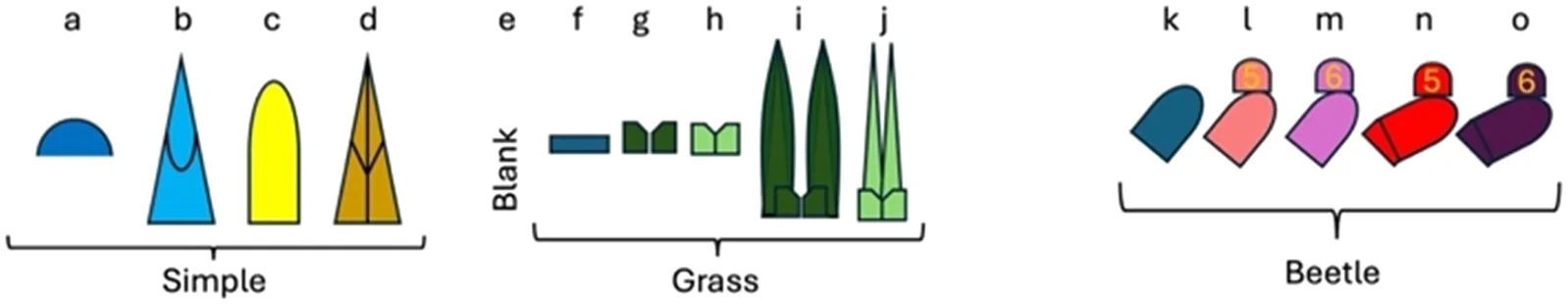
2D versions of models a–o (a) hemispheres, (b) cones, (c) paraboloids, (d) pyramids, (e) blank (just a Petri dish with no design), (f) block base of grass designs with no channels, (g) 4 × 4 base of spike design, (h) 6 × 6 base of spike design, (i) 4 × 4 grass inspired spike design, (j) 6 × 6 grass inspired spike design, (k) beetle back base design with no channels, (l) 5 bumps across flat beetle design, (m) 6 bumps across flat beetle design, (n) 5 bumps across bent beetle design, and (o) 6 bumps across bent beetle design.

For easier comparison, the 2D versions' summary of all the designs has been used throughout the rest of this paper, which is shown in Fig. 8, as well as the physical images of the 3D printed models which are shown in Fig. S3.

### 3.4. Comparison of superhydrophobic and superhydrophilic coatings designs

**Fig. 9:**
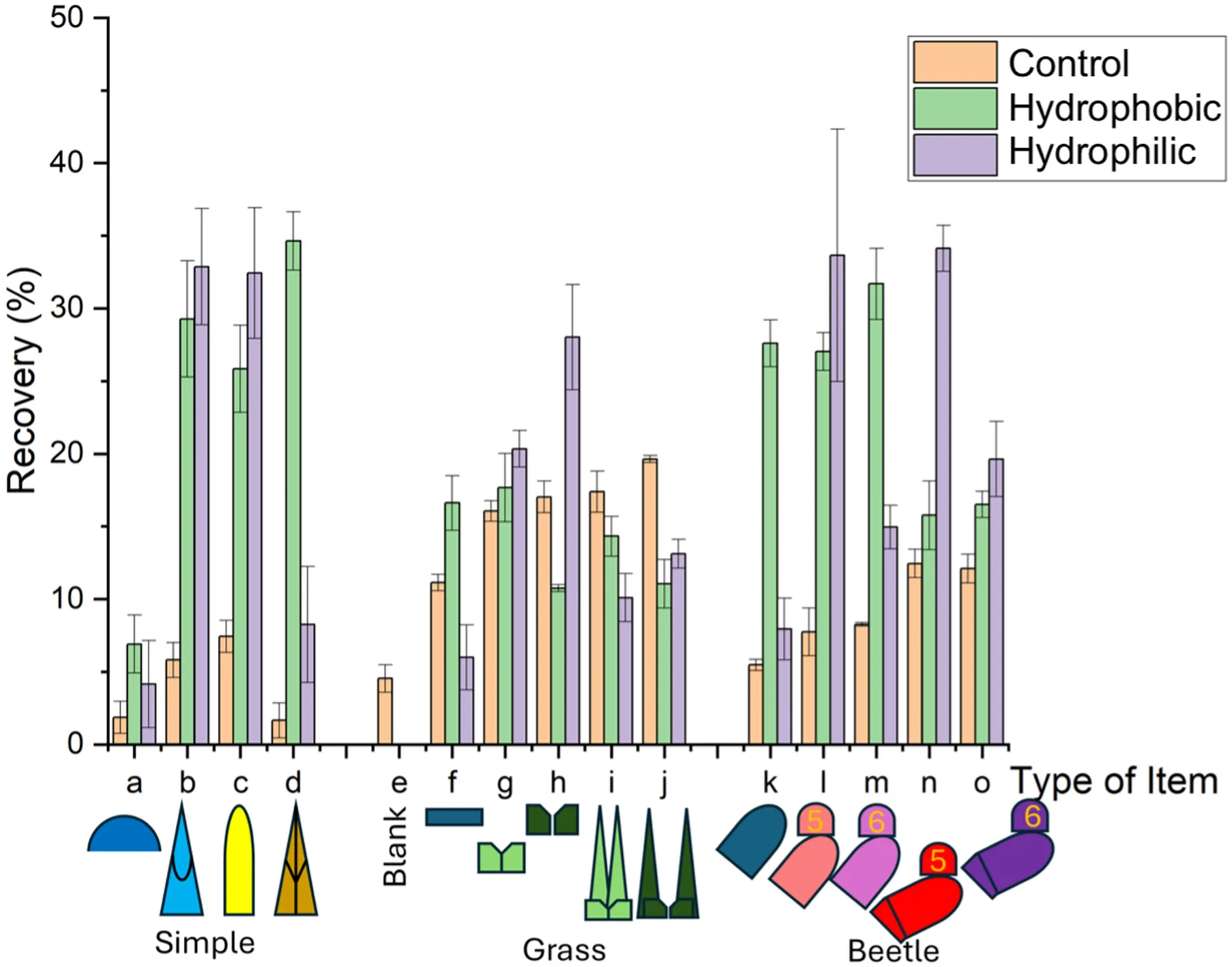
A graph of water recovery for models (a–o), uncoated, coated with hydrophobic and hydrophilic nanoparticles. *N* = 3.

The three categories of designs were tested uncoated as a control, with the only variation being the surface area and the shapes themselves (simple shapes, grass-inspired, and beetle-inspired designs). Temperature and humidity were recorded to monitor any environmental variations, and water recovery was measured. Detailed data are presented in Table S2 By comparing the initial samples in Table S2 with the water recovery percentages shown in Fig. 9, it is evident that the taller shapes—designs (b) and (c) (cone and paraboloid)—achieved higher water collection (∼6–7%) than design (a), the hemispheres (<2%). This is likely due to their larger surface area and smoother surfaces, which facilitate droplet formation. In contrast, the pyramid shape (d) collected less water (∼2%), which we believe is due to its sharper vertices and edges. These features reduce the smooth surface area available for droplet attachment, thereby hindering water collection.^44^

The control results for the Namib grass-inspired designs tell a similar story. The Petri dish (e) alone recorded the lowest water recovery (∼3%), while the two models (i and j) showed increased recovery (18–20%) with greater design complexity—and consequently, increased surface area. This improvement is likely due to the sloped surfaces of the models, which facilitate condensation as fog passes over them.^43,45,46^ Notably, the fogged water volume was more variable in this case, as the testing duration was adjusted to determine optimal conditions.

The experiment was repeated with the entire surface of each model coated in hydrophobic nanoparticles to assess the impact of wettability on water runoff. Wettability had a substantial effect, significantly increasing recovery percentages across all models. As shown in Fig. 9 and Table S3, the simple models, once coated, outperformed their uncoated counterparts by a factor of 3–4 fold, while maintaining a similar performance ranking. The hemisphere model (a) continued to perform poorly compared to the taller cone (b), paraboloid (c), and pyramid (d) shapes. Interestingly, model (c) did not show as large an increase in water recovery as models (b) and (d), likely because its flattened vertex is less effective at gathering droplets than the sharper, more pointed features of the other designs.

The grass-inspired designs present an interesting case: the spiked samples, models (i and j), recorded lower water recovery values after coating compared to their uncoated counterparts. The exception is model (f), the control square, which showed a higher recovery. This is likely due to its initially poor collection performance, although it still exhibits high droplet mobility once droplets form. In contrast, the groove-structured models (g and h) did not experience as significant a reduction in performance as the spiked samples. A plausible explanation for the poor performance of the spiked designs under hydrophilic conditions is that they are the only models featuring sideways channels. These lateral pathways may trap droplets, preventing them from moving downward into the collection trough. On the other hand, the flat-based and beetle-inspired designs feature only vertical channels, which promote droplet mobility *via* gravity once the surface becomes saturated.^20,44^

The beetle-inspired designs showed an overall increase in water recovery across all samples. However, the flat models (k, l, and m) exhibited a significantly greater improvement in water collection compared to their uncoated versions. In contrast, the curved models (n and o) were less effective at gathering and mobilising droplets. This is likely because a large portion of their surface area does not directly face the incoming fog, reducing their ability to intercept and redirect it effectively. As a result, these curved surfaces are less capable of initiating droplet formation and guiding water toward the collection trough.^7^

For samples coated with superhydrophilic particles, the smoother-edged shapes, cone and paraboloid (models b and c), which lack vertices, performed better than their uncoated counterparts (see Table S4 and Fig. 9). This improvement is attributed to the avoidance of the “star-stepping” effect, which can hinder water collection.^42^ In contrast, the pyramid design (d), with its sharp vertices, showed a significant reduction in water recovery (8%), while the hemisphere model (a) was less effective at mobilising water compared to when it was coated with hydrophobic particles.

Among the grass-inspired designs, the flat square model (f) performed poorly, failing to direct collected water efficiently into the collection dish. The spiked designs benefited from increased surface area, collecting more water than their flat counterparts. However, the lower-density spike model (i, with a 4 × 4 spike array) was less effective at mobilising the collected fog. This is likely due to droplets becoming trapped in the vertical channels, preventing them from draining into the collection trough.^44^ For the beetle-inspired designs, the flat control model (k) was less effective at collecting droplets than when coated with hydrophobic particles. In contrast, the lower-density, larger-bump models (l and n) performed significantly better (∼34%) than the higher-density, smaller-bump models (m and o), which achieved only ∼15–19%. The larger features appear to attract and retain more water, enhancing collection efficiency. Our experiments indicate that excessively dense arrays can impede coalescence and reduce droplet mobility, leading to pinning within narrow lateral channels or crevices and, consequently, lower net drainage. Larger spikes/bumps offered a more favourable balance of capture, coalescence, and shedding, yielding higher overall collection rates under our conditions.

### 3.5. Mixed hydrophilic/hydrophobic coatings

**Fig. 10:**
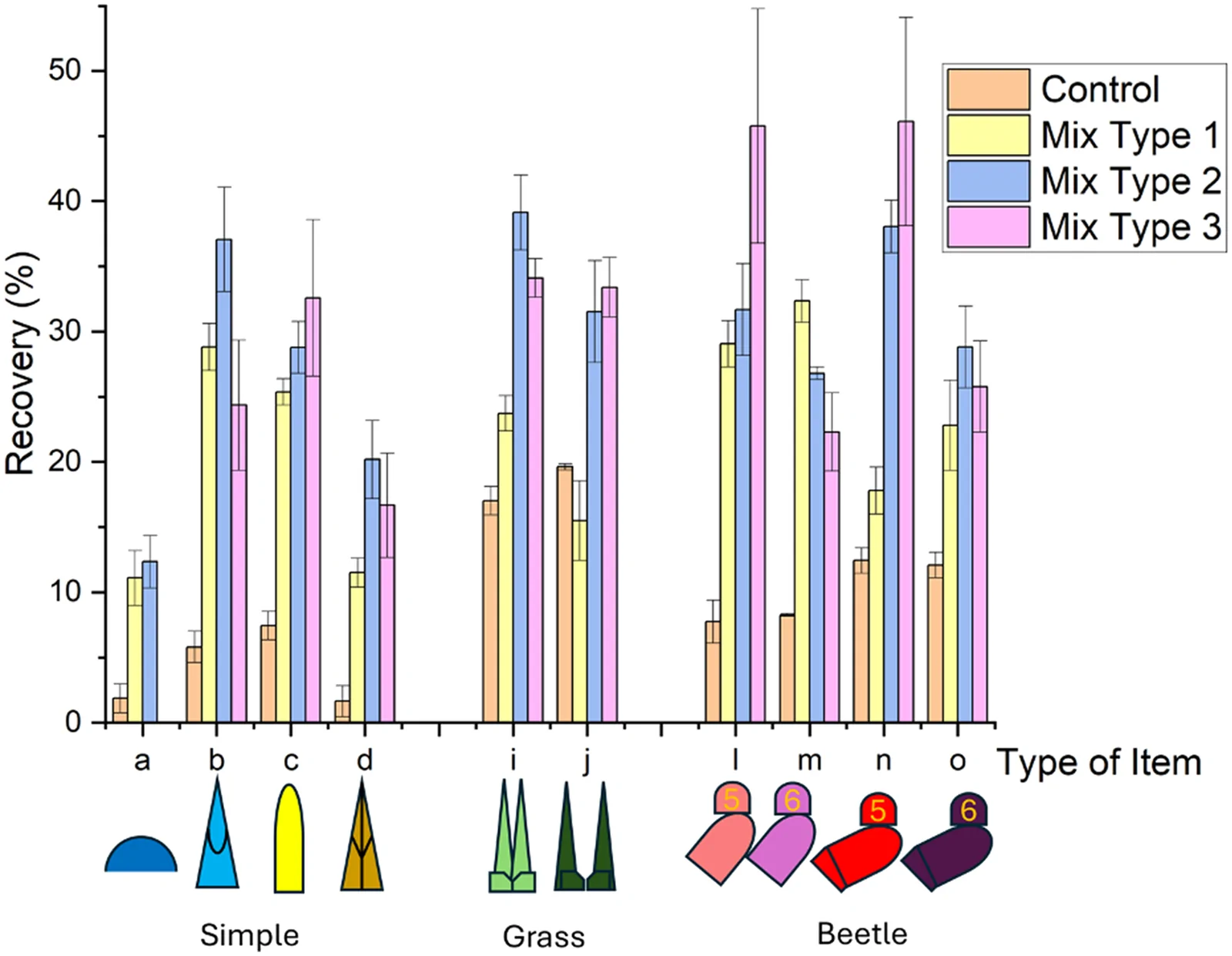
A graph of water recovery for models (a–o), with coated spikes of type 1 (100% hydrophilic), type 2 (50% hydrophilic & 50% hydrophobic), and type 3 (75% hydrophilic & 25% hydrophobic), please note that for all samples the base of the structure is coated with a hydrophobic coating to encourage droplet mobility for collection. *N* = 3.

Further study into the water recovery of the designs was conducted through coating discrete areas of the specimens with one type of nanoparticles and then coating the rest of the sample with the other type (Fig. 2). This increase in complexity was inspired by the Namibian desert beetle, which utilises both hydrophilic and hydrophobic structures in its water collection process. Three coating combinations were tested. In type 1, the base was coated with hydrophobic particles, while the spikes or bumps were fully hydrophilic (see Table S5 and Fig. 10). Under this configuration, both the hemisphere and cone designs (models a and b) recorded high water recovery percentages, with the paraboloid and pyramid models (c and d) also performing well. This enhanced performance is likely due to the synergistic effect of combining hydrophilic and hydrophobic regions. The hydrophilic spikes effectively capture fog droplets, which condense and spread across the surface. As the droplets accumulate, they are guided downward by gravity and eventually reach the hydrophobic base. There, the low surface energy promotes droplet beading and rolling, allowing the water to efficiently drip into the collection container.^47^

The grass-inspired models (i and j) exhibited notable performance under hybrid coating conditions. Model (i), characterised by a higher spike density, demonstrated higher efficiency at ∼24%, while Model j achieved a water collection efficiency of ∼15%.

In contrast, the beetle-inspired designs (models l–o) showed more varied results. The larger bump configurations (models l and n) performed suboptimally, with water collection efficiencies of ∼29% and ∼17%, respectively. However, models (m) and (o), which featured higher bump densities, yielded improved results, achieving efficiencies of ∼32% and ∼23%, respectively.

This suggests that bump density plays a critical role in enhancing water collection, specifically under type 1 hybrid coating conditions, where smaller, more densely packed features appear to be more effective at attracting and retaining droplets. This trend, however, does not hold consistently across other coating types.

In the type 2 configuration, where both hydrophilic and hydrophobic particles were applied to the spike/bumps in a 50 : 50 ratio, a precise masking line was used halfway up each feature (as illustrated in Fig. 2). The results, shown in Table S6 and Fig. 9, indicate that all samples exhibited improved water collection compared to both their uncoated versions and those coated using the type 1 method. Notably, the hemisphere (a) and cone (b) designs achieved record-high recovery rates, with the paraboloid (c) and pyramid (d) models also performing strongly.

The grass-inspired designs (i and j) also showed high collection efficiencies, reaching ∼40% and ∼31%, respectively. Interestingly, the beetle-inspired models (l–o) performed particularly well under this coating strategy, with the larger bump configurations (l and n) outperforming the smaller, higher-density models (m and o), a reversal of the trend observed in type 1. This suggests that the effectiveness of bump density is highly dependent on the specific hydrophobic/hydrophilic coating configuration.

Type 3 samples were also tested, with results presented in Table S7. Design (a) was excluded due to the difficulty of applying a precise masking line to its geometry. Building on the promising results from the mixed-coated spikes in type 2, type 3 introduced a coating ratio of approximately ¾ hydrophilic to ¼ hydrophobic along the spike surfaces.

Under this configuration, the paraboloid design (c) performed best among the simple shapes, achieving a water recovery of 32%. However, the cone and pyramid models (b and d) showed a slight decrease in performance (3–10% lower than their type 2 counterparts), though they still outperformed most other coating types.

The grass-inspired designs (i and j) delivered excellent results, with both achieving around 34% water recovery, likely due to their longer spike spacing, which facilitates droplet mobility. The beetle-inspired designs with larger bumps (l and n) recorded the highest recovery rates of all samples, reaching 45% and 46%, respectively—an increase of approximately 32% and 70% compared to type 2 and type 1 coatings. In contrast, models with smaller bumps (m and o) showed reduced water collection efficiencies of 22% and 25%, consistent across all three coating types.

The compromise between hydrophilic and hydrophobic areas arises from the competing requirements of droplet nucleation and droplet transport. Hydrophilic regions promote rapid nucleation and growth, whereas hydrophobic regions are essential for droplet mobility and shedding. Consequently, increasing the hydrophilic area fraction does not necessarily enhance water collection, as excessively large hydrophilic zones can lead to droplet pinning and reduced shedding efficiency. The variations observed in Fig. 10 indicate that performance depends not only on the overall hydrophilic fraction but also on the geometry and spatial distribution of these regions. The optimal designs (types 2 and 3) combine relatively large hydrophilic areas (50–75%) with sharp, continuous hydrophobic pathways that facilitate efficient droplet removal (Fig. S1), consistent with the microstructural features seen in the SEM images of the rice leaf (Fig. S2). Thus, efficient water harvesting is governed by the interplay between nucleation density and droplet transport rather than hydrophilic area alone.

Overall, these findings illustrate the importance of both geometry and wettability in determining water-collection performance, as is also evident in natural systems. The results suggest that larger bumps with greater surface area are more effective for water harvesting. In contrast, the effects of feature size and density are less clear for the spike geometries, as their water-collection efficiency is strongly influenced by the presence of sideways, mesh-like channels. Nevertheless, the hybrid coating conditions and the specific hydrophilic-to-hydrophobic ratio play a critical role in optimising performance across all geometries studied here.

The wide variability in efficiencies reported in the literature reflects the strong dependence of fog-harvesting performance on substrate chemistry, hierarchical structuring, and environmental conditions, as shown in Table 1. While some micro/nanostructured surfaces achieve exceptionally high laboratory-scale rates, these often rely on specialised materials or controlled flow environments that limit direct comparison and real-world applicability. For example, hierarchical micro/nanostructures with wettability control have reported rates in the hundreds of mg cm^−2^ h^−1^ to several g cm^−2^ h^−1^ under controlled fog flows, substantially exceeding simple meshes.^48,49^ In contrast, field scale systems (*e.g.*, standard fog collectors) typically yield 1–10 L m^−2^ day^−1^, with strong site dependence.^50^ Techno economic analyses emphasize gaps between laboratory performance and outdoor robustness/scale.^51^

**Table 1:** Summary of reported studies highlighting how changes in geometry, substrate type, and surface wettability influence water-collection performance

Study	Substrate material	Design topography	Coating applied	Maximum collection efficiency g cm^−2^ h^−1^
Xiao *et al.*^48^	Photocurable diacrylate/polyolefin resin	Conical spine, gradient microgrooves and oriented thorns	None	5.9
Yin *et al.*^52^	Copper	Mesh on flat sheet	PTFE nanoparticles	0.2
Zhu *et al.*^53^	Copper	Flat sheet	TiO_2_/hydrophobic Cu nanoparticles	1.3
Qadir *et al.*^50^	Fabric	Net	None	5.2

In contrast, our study isolates the geometric and wettability-patterning parameters under controlled conditions, enabling clearer mechanistic insight into how shape and hybrid wetting interact to influence droplet nucleation and transport. This positions our work as a foundational design study that complements, rather than competes with, high-performance biomimetic systems by identifying the specific geometric features and hydrophilic–hydrophobic distributions that most effectively enhance passive water collection.

## 4. Conclusions

This study demonstrates that fog-water harvesting performance is governed not by a single structural feature but by the synergistic interplay between surface geometry and wettability patterning. By drawing inspiration from three highly efficient natural collectors, the Namib desert beetle (*Stenocara gracilipes*), the dune bushman grass (*Stipagrostis sabulicola*), and the microtextured leaf of *Oryza sativa*, we designed biomimetic 3D-printed surfaces and enhanced them with hybrid hydrophilic–hydrophobic nanoparticle coatings. These engineered surfaces successfully captured water from both fog and dew, translating biological strategies into scalable synthetic designs.

Across all geometries, mixed-wettability coatings significantly outperformed uncoated and fully hydrophobic/hydrophilic coated controls, confirming that combining hydrophilic nucleation sites with hydrophobic transport pathways is essential for efficient collection. Type 2 coatings (50 : 50 hydrophilic–hydrophobic) delivered the most reliable improvements across diverse structures, while type 3 coatings (¾ hydrophilic–¼ hydrophobic) further boosted efficiency in specific morphologies. In particular, the beetle-inspired models with larger, well-spaced bumps achieved the highest measured efficiency of up to 46%, illustrating the strong coupling between feature size, surface chemistry, and transport dynamics.

Geometry itself proved equally critical. Taller, smoother-edged structures, such as cones and paraboloids, facilitated more effective droplet flow, especially when paired with hybrid coatings. Grass-inspired arrays benefited from increased surface area and optimized spike spacing, whereas beetle-inspired textures revealed that the interplay between bump density and the coating strategy can either enhance or hinder collection depending on the balance of nucleation and shedding.

Overall, our findings highlight that high-performance fog harvesting requires the deliberate integration of geometry, texture, and spatially distributed wettability. This work provides a clear design framework for next-generation passive water-collection devices and demonstrates how biological principles can be translated into engineered solutions capable of operating efficiently in arid and fog-rich regions.

## Author contributions

Henry Apsey: methodology, validation, formal analysis, investigation, resources, writing – original draft, visualization. Donald Hill: review & editing, writing. Shirin Alexander: conceptualization, validation, resources, writing, review & editing, supervision, project administration, funding acquisition.

## Conflicts of interest

There are no conflicts to declare.

## Supplementary Material

LF-003-D5LF00222B-s001

## Data Availability

All data supporting this study are available within the article and its supplementary information (SI). Additional raw/processed data required to reproduce these findings can be obtained from the corresponding author upon request. Supplementary information: water collection data for all the designs and coatings. SEM images of the rice leaves. Masking and coating images of the hybrid system. Videos showing the sharp interface between the two coatings. See DOI: https://doi.org/10.1039/d5lf00222b.
