# Correction: Biomimetic engineering for water harvesting: 3D printed solutions for arid regions

**DOI:** 10.1039/d6lf90018f

**Published:** 2026-07-21

**Authors:** Henry Apsey, Donald Hill, Shirin Alexander

**Affiliations:** a School of Engineering and Applied Sciences, Department of Chemical Engineering, Swansea University Swansea Wales UK s.alexander@swansea.ac.uk

## Abstract

Correction for ‘Biomimetic engineering for water harvesting: 3D printed solutions for arid regions’ by Henry Apsey *et al.*, *RSC Appl. Interfaces*, 2026, **3**, 776–786, https://doi.org/10.1039/d5lf00222b.

The authors regret labelling errors in Fig. 4, 5 and 8 and associated errors in the main text in the original article. The authors now provide the correctly labelled figures and text clarifications and confirm that these changes do not affect the conclusions presented.

**Fig. 4:**
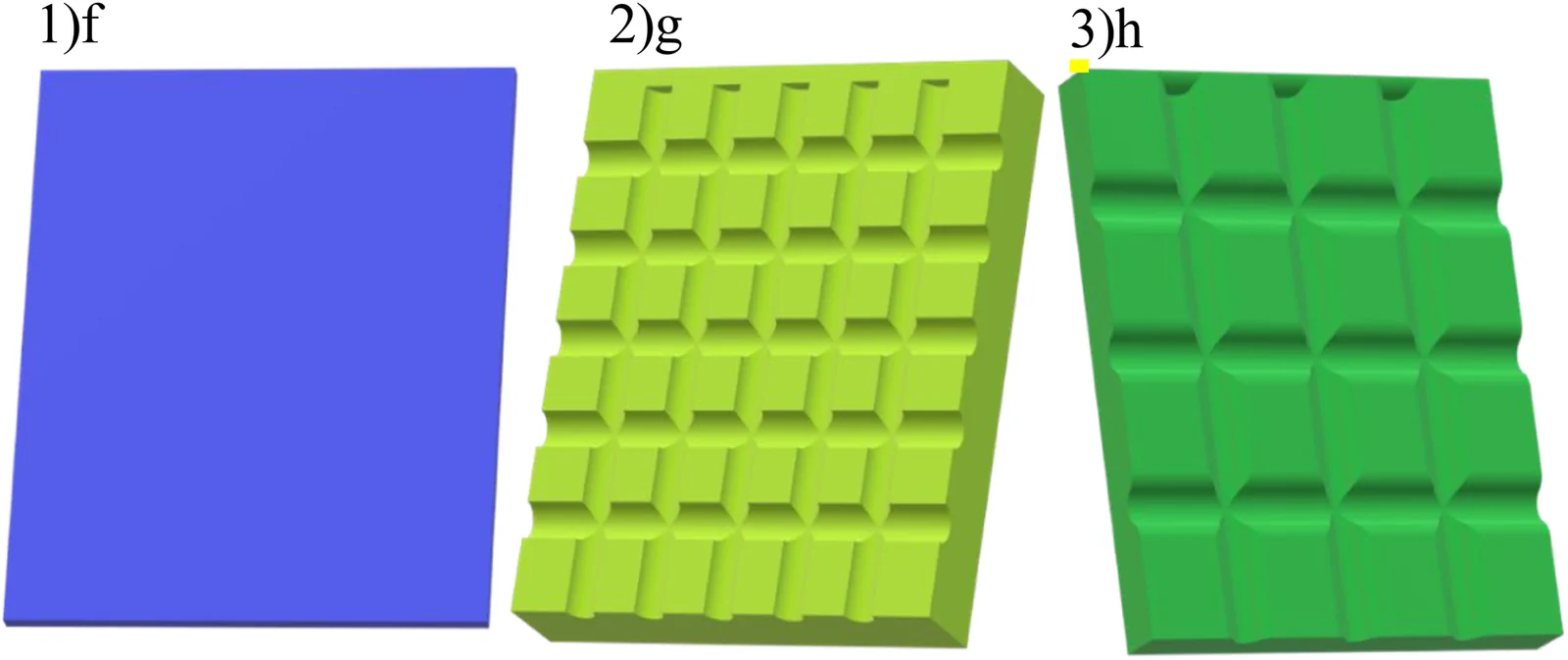
A set of control designs inspired by grass spikes. 1) Design (f), a simple smooth base used as a control block of PLA, 2) design (g), a control base of the 6 × 6 spike design without the spikes, 3) design (h), a control base of the 4 × 4 spike design without the spikes.

The text associated with Fig. 4 in section 3.3.2. states that design (f) was compared with designs (g) (channels of 4 × 4) and (h) (channels of 6 × 6) to examine the impact of the channels, and their density without spikes. Considering the corrected Fig. 4 here, design (g) has channels of 6 × 6, and design (h) has channels of 4 × 4.

**Fig. 5:**
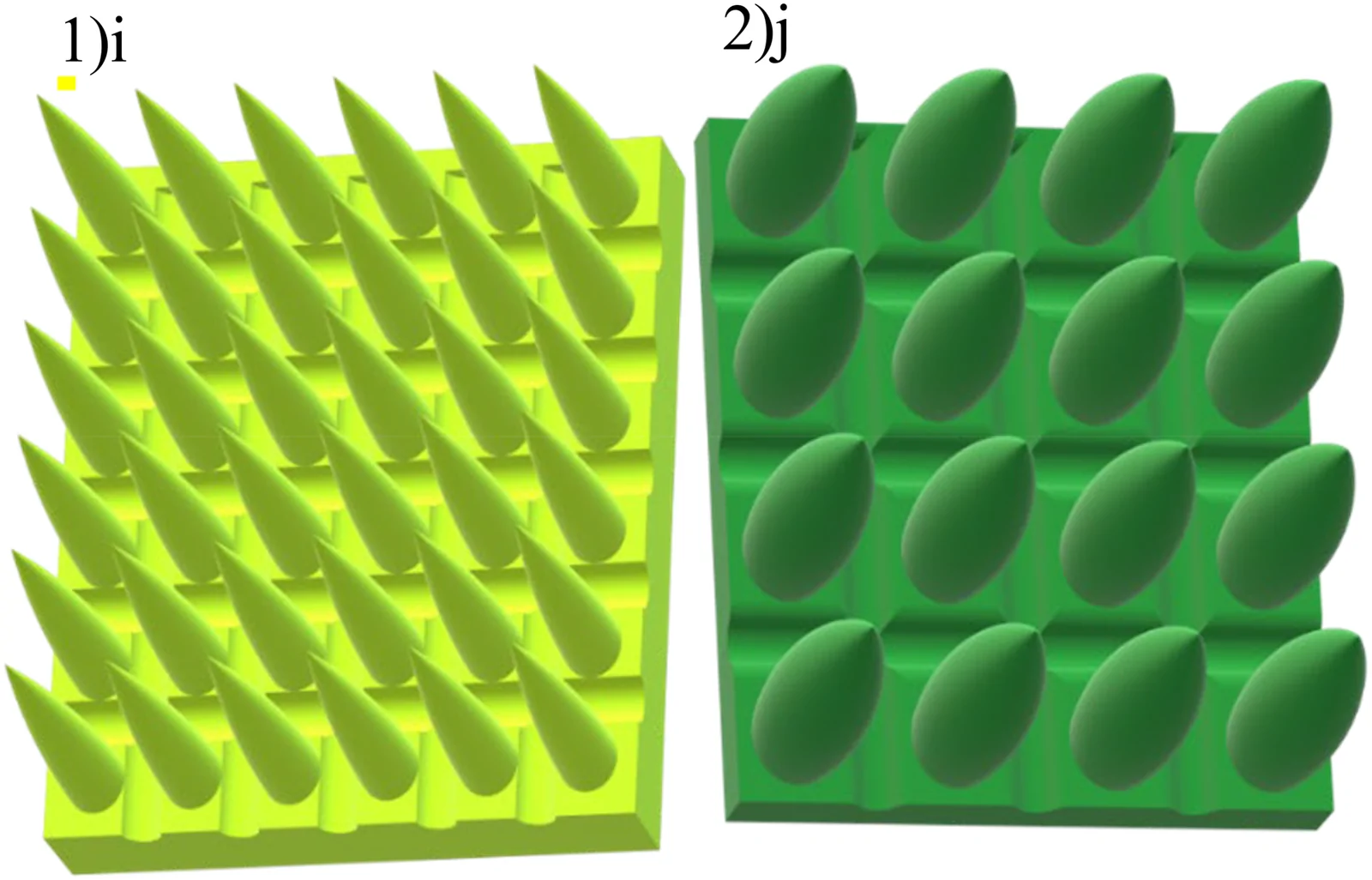
A set of designs inspired by grass spikes. 1) Design (i) a high-density grass-inspired design with 6 spikes across the substrate & 2) design (j) a low-density grass-inspired design with 4 spikes across the substrate.

The text associated with Fig. 5 in section 3.3.2. states “In a design of 15 × 15 cm, it was chosen to test a 4 × 4 design (i) and a 6 × 6 spikes design (j) of the same height, as shown in Fig. 5.” Considering the corrected Fig. 5 here, design (i) has a 6 × 6 design and design (j) has a 4 × 4 design.

**Fig. 8:**
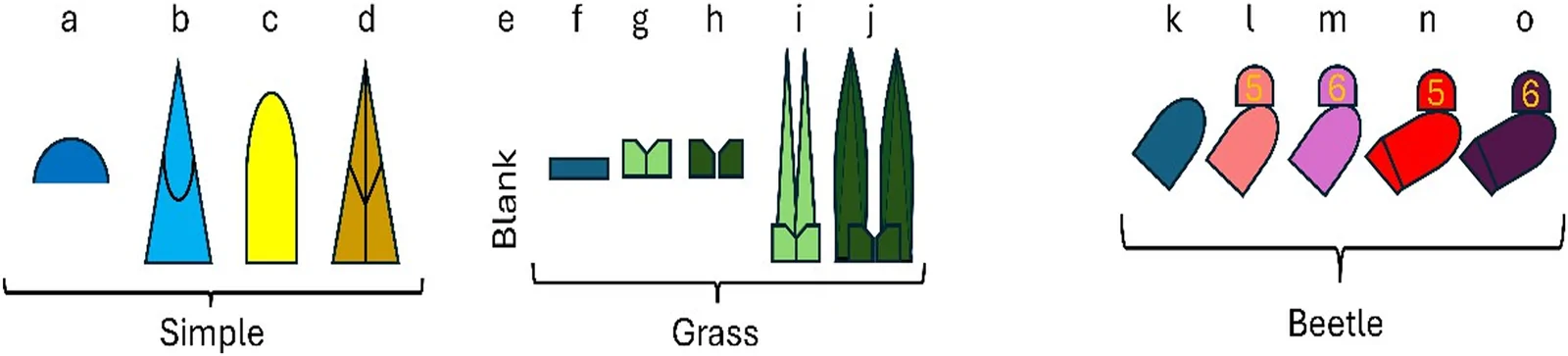
2D versions of models a–o: (a) hemispheres, (b) cones, (c) paraboloids, (d) pyramids, (e) blank (just a Petri dish with no design), (f) block base of grass designs with no channels, (g) 6 × 6 base of spike design, (h) 4 × 4 base of spike design, (i) 6 × 6 grass inspired spike design, (j) 4 × 4 grass inspired spike design, (k) beetle back base design with no channels, (l) 5 bumps across flat beetle design, (m) 6 bumps across flat beetle design, (n) 5 bumps across bent beetle design, and (o) 6 bumps across bent beetle design.

The text associated with Fig. 8 in section 3.4. states “the lower-density spike model (i, with a 4 × 4 spike array) was less effective at mobilising the collected fog”. Considering the corrected Fig. 8 here, the higher-density spike model (i, with a 6 × 6 spike array) should be considered to be less effective at mobilising the collected fog.

The Royal Society of Chemistry apologises for these errors and any consequent inconvenience to authors and readers.

