# Supplementary material for: Biomimetic engineering for water harvesting: 3D printed solutions for arid regions
Source: RSC Appl Interfaces. 2026 Mar 4;3(3):776–86. doi: 10.1039/d5lf00222b (PMC13584820; doi:10.1039/d5lf00222b)
Supplement: Supplementary file 1 [file LF-003-D5LF00222B-s001.pdf]

Supplementary Information (SI) for RSC Applied Interfaces.
This journal is © The Royal Society of Chemistry 2026

Supporting information for:

Biomimetic Engineering for Water Harvesting: 3D Printed Solutions for Arid Regions

Henry Apsey,[1] Donald Hill,[1] Shirin Alexander *[1]

[1]*School of Engineering and Applied Sciences, Department of Chemical Engineering, Swansea University, Swansea, Wales, UK.*

SI Methods Additional detail

SEM of rice leaves:

The *Oryza sativa* rice leaf was mounted on an aluminium SEM stub via a carbon tab and sputter coated with copper to improve conductivity using a Quorum Q150T S. Scanning electron microscopy images were acquired using a Carl Zeiss Crossbeam 550L (Zeiss, Germany) operated in secondary electron mode, with an accelerating voltage of 5 kV, a probe current of 500 pA, and a working distance of 5.1 mm.

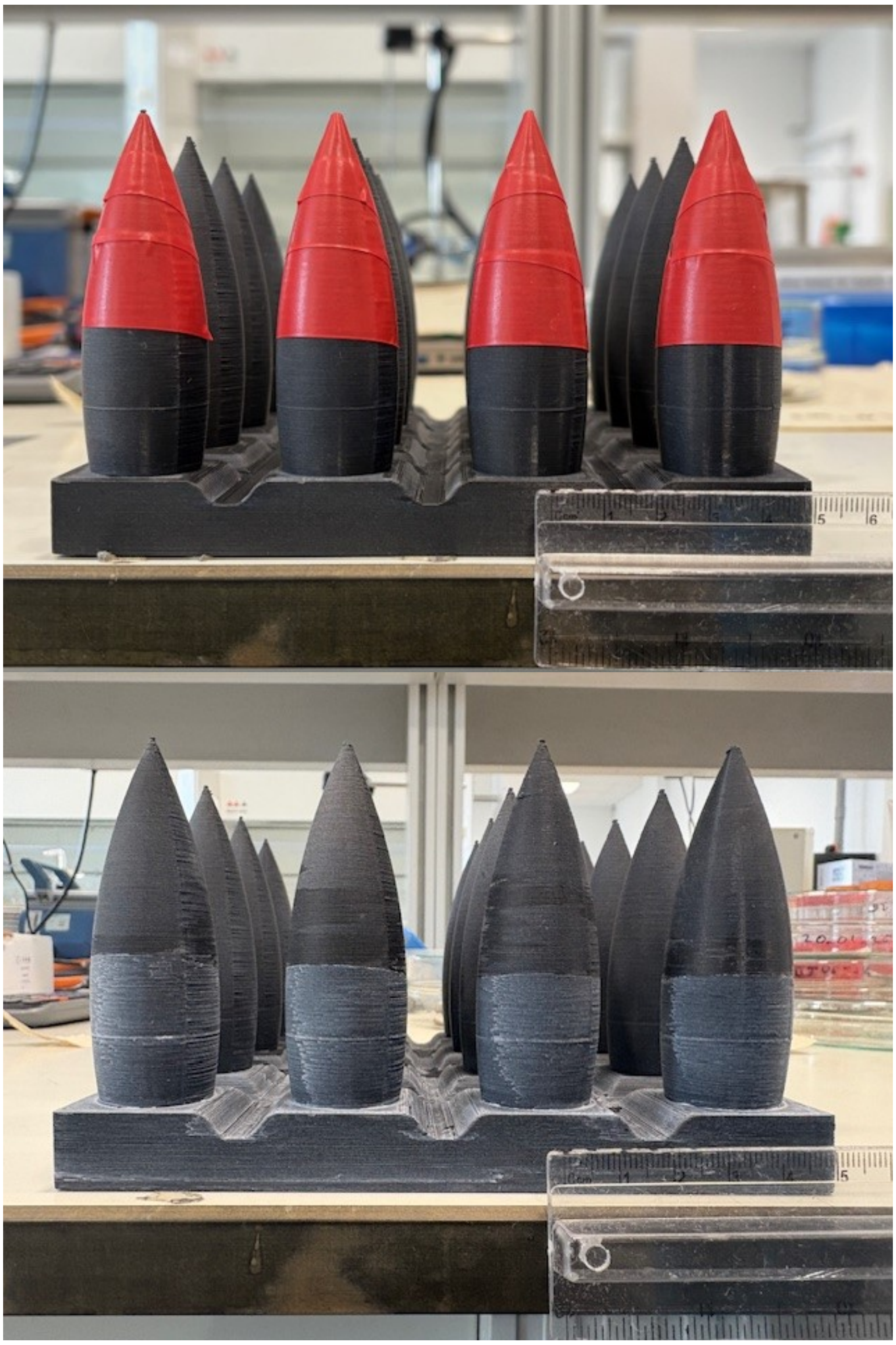

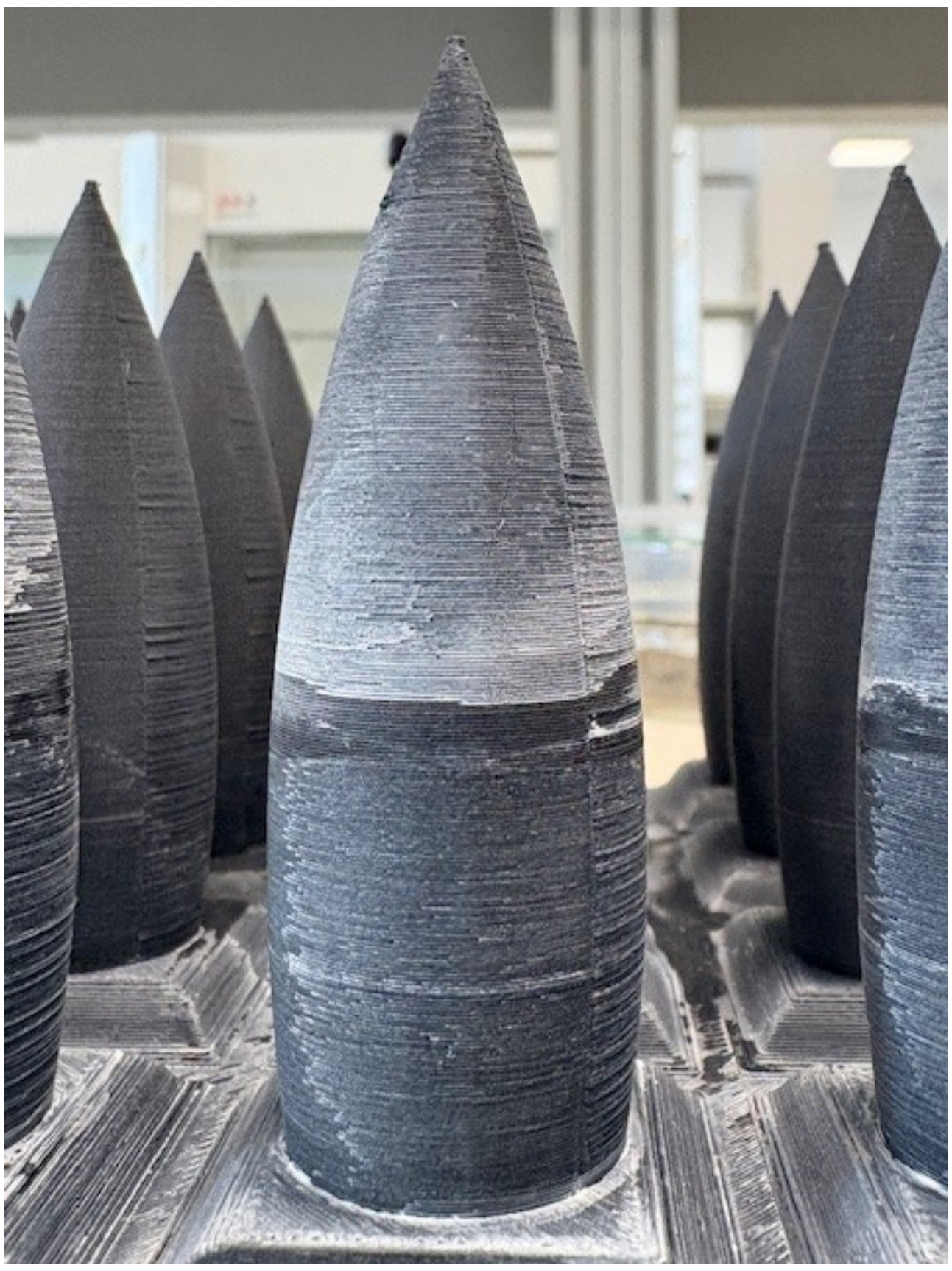

https://www.icloud.com/iclouddrive/0f0EZxN82fqI-jjwEov3lGnPg#IMG_1240

https://www.icloud.com/iclouddrive/02aN5zuxpmmsKTjEtpQ39xkbA#IMG_1238

Figure S1: Photographs of the masking for the 50:50 as a model system (50% of the spike hydrophilic, the rest and the base hydrophobic, Type 2), as well as the videos showing precise, sharp masking lines with clearly defined boundaries between the hydrophobic and hydrophilic coatings.

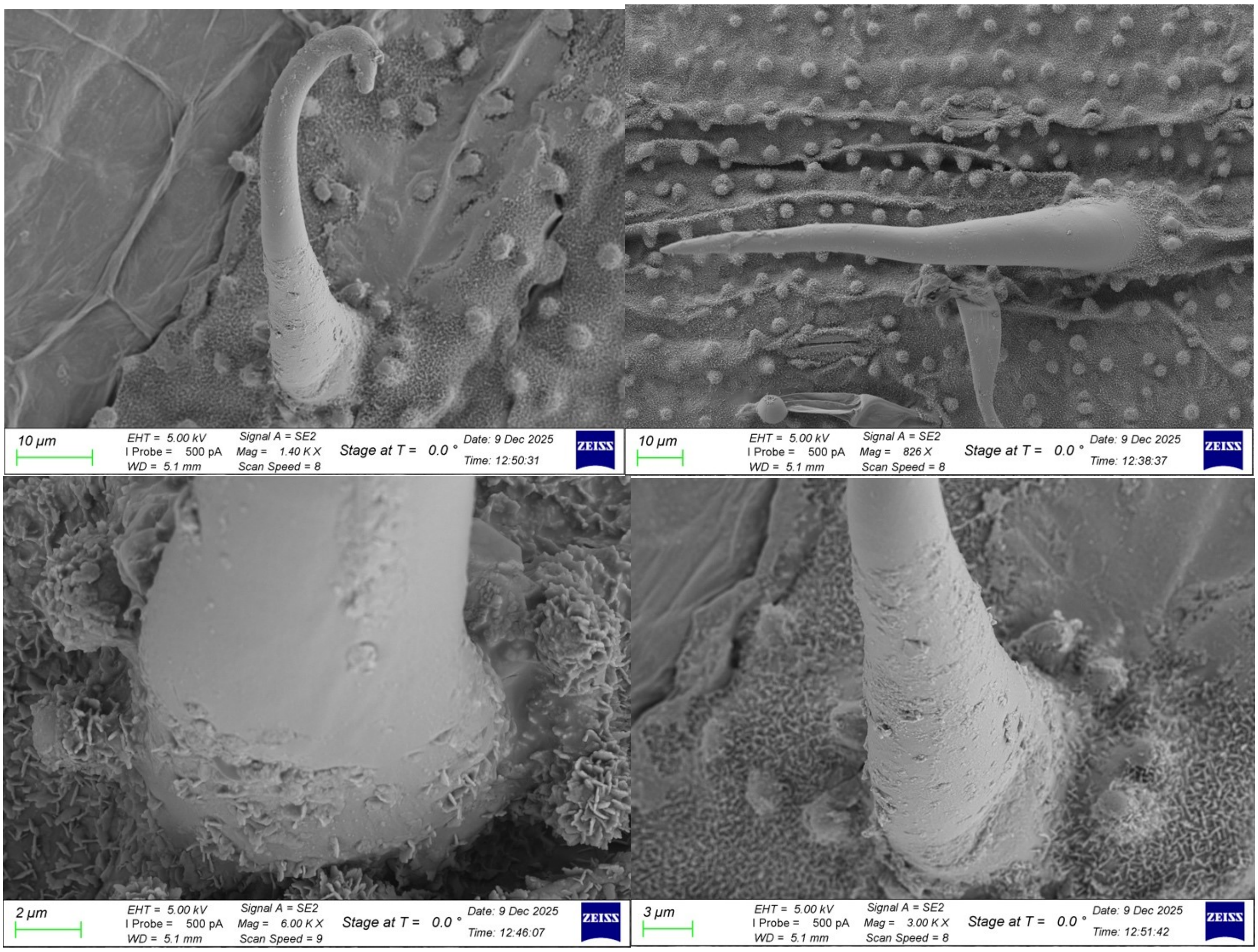

Figure S2: SEM micrograph of the inspiration rice leaf (Oryza Sativa) structure showing its array of wax structures.

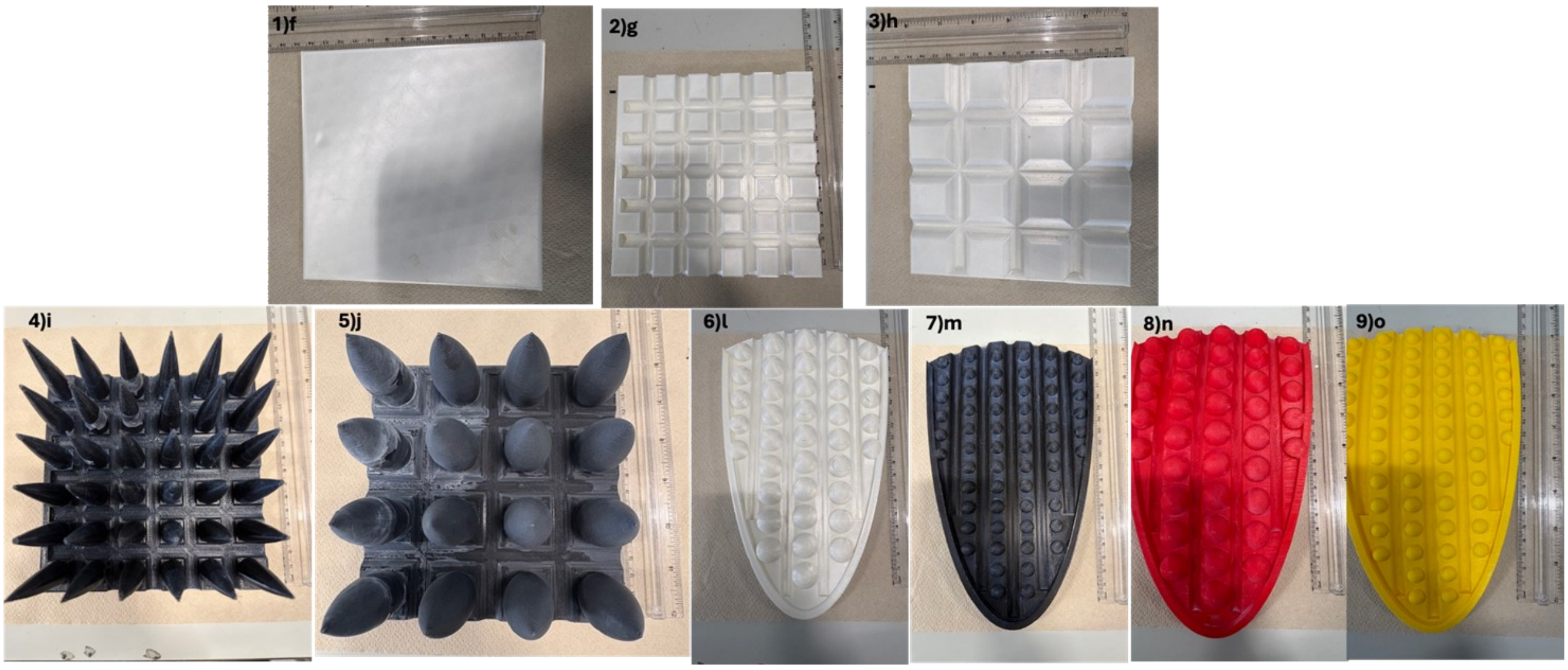

Figure S3. Physical images of 3D printed models: (1-f) block base of grass designs with no channels, (2-g) 6x6 base of spike design, (3-h) 4x4 base of spike design, (4-i) 6x6 grass inspired spike design, (5-j) 4x4 grass inspired spike design, (6-l) 5 bumps across flat beetle design, (7-m) 6 bumps across flat beetle design, (8-n) 5 bumps across bent beetle design, and (9-o) 6 bumps across bent beetle design.

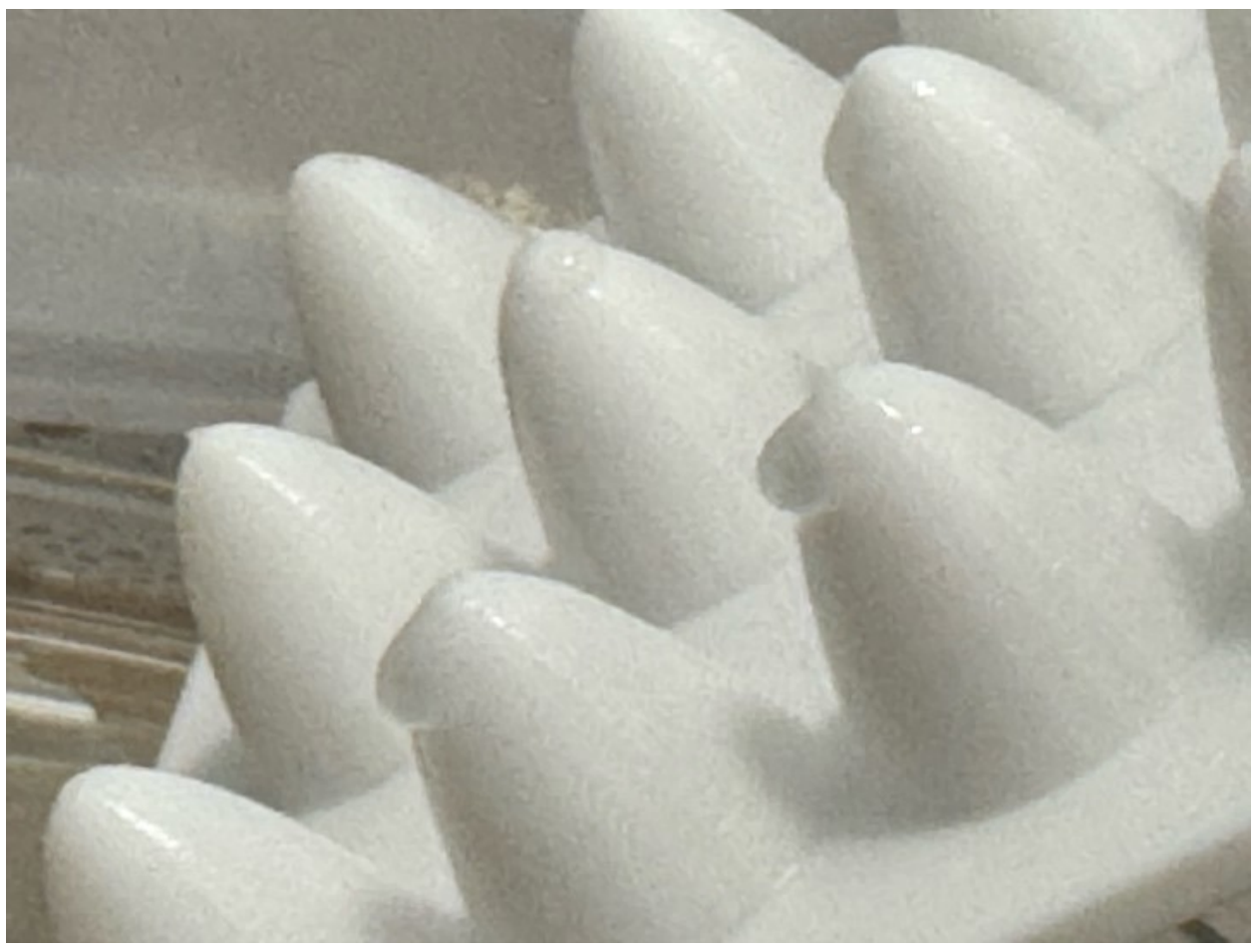

Figure S4. Photo of sampels showing droplet formation on the end of bump structures.

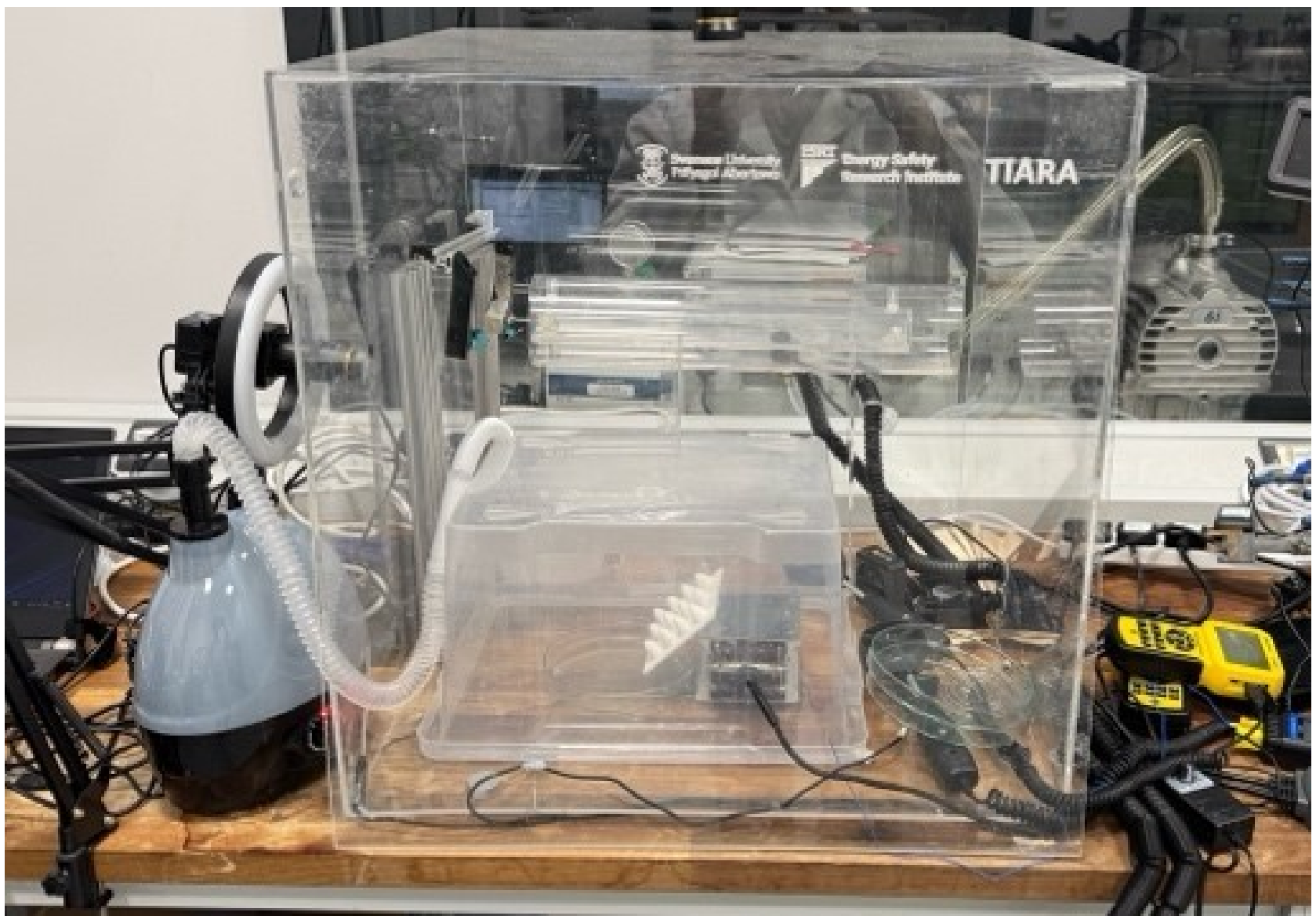

Figure S5. Photo of the climate chamber used for the water collection experiment.

Table S1. Contact Angles for different types of 3D print plastic upon a flat printed surface.

	Contact Angle /º		
Item	Uncoated	Coated with Hydrophilic Al_2O_3 Particles	Coated with Hydrophobic Al_2O_3 Particles
PLA Filament	11.6 ±4.7	<1	155.7±12.6
ABS Filament	12.3±10.1	<1	156.2±10.4
PETG Filament	14.8±11.2	<1	153.4±11.8
SLA Resin	25.4±11.4	<1	150.1±14.5

Table S2: Water collection data for uncoated samples. Variation is based on 3 replicates of full-cycle water collection.

Item	Recovery / %	Variation	Surface area / mm^2
a) Hemisphere	1.86	±1.12	8536
b) Cone	5.80	±1.22	9043
c) Paraboloid	7.43	±1.16	9719
d) Pyramid	1.65	±1.25	9349
e) Petridish Only	3.20	±0.95	N/A
f) Flat Square	10.43	±0.56	4800
g) Groove Square 6	16.27	±0.71	56325
h) Groove Square 4	17.75	±1.09	65527
i) Spikes 6	18.87	±1.41	136486
j) Spikes 4	19.42	±0.23	153505
k) Control Base D	5.07	±0.39	56160
l) D Flat 5	8.40	±1.64	67729
m) D Flat 6	8.32	±0.12	64654
n) D Curve 5	12.35	±0.98	68160
o) D Curve 6	12.48	±0.99	64752

Table S3: Water collection for samples coated with superhydrophobic Al_2O_3 Nanoparticles.

Item	Recovery / %	Variation
a) Hemisphere	6.90	±2.01
b) Cone	29.27	±4.12
c) Paraboloid	25.85	±3.27
d) Pyramid	34.64	±2.32
f) Flat Square	16.61	±1.88
g) Groove Square 6	17.66	±2.35
h) Groove Square 4	11.05	±0.20
i) Spikes 6	14.70	±1.50
j) Spikes 4	11.10	±1.67
k) Control Base D	27.59	±1.62
l) D Flat 5	27.02	±1.30
m) D Flat 6	31.69	±2.45
n) D Curve 5	15.76	±2.35
o) D Curve 6	16.50	±0.91

Table S4: Water collection for samples coated with superhydrophilic Al_2O_3 Nanoparticles

Item	Recovery / %	Variation
a) Hemisphere	4.16	±3.34
b) Cone	32.86	±3.75
c) Paraboloid	32.44	±4.50
d) Pyramid	8.25	±4.04
f) Flat Square	5.99	±2.24
g) Groove Square 6	20.34	±1.27
h) Groove Square 4	28.01	±3.62
i) Spikes 6	10.10	±1.65
j) Spikes 4	13.12	±0.99
k) Control Base D	7.94	±2.13
l) D Flat 5	33.65	±0.68
m) D Flat 6	14.95	±1.50
n) D Curve 5	34.13	±1.58
o) D Curve 6	19.63	±2.59

Table S5: Water collection for samples coated with a mix of superhydrophilic (spikes)/ superhydrophobic (base) Al_2O_3 Nanoparticles (Type 1).

Item	Recovery / %	Variation
a) Hemisphere	11.11	±2.14
b) Cone	28.82	±1.88
c) Paraboloid	25.37	±1.67
d) Pyramid	11.51	±1.51
i) Spikes 6	23.71	±1.35
j) Spikes 4	15.49	±3.06
l) D Flat 5	29.07	±1.77
m) D Flat 6	32.35	±1.63
n) D Curve 5	17.81	±1.81
o) D Curve 6	22.81	±3.45

Table S6: Water collection for samples coated with a 50/50% mix (upon the spikes) of superhydrophilic/ superhydrophobic Al_2O_3 Nanoparticles (Type 2).

Item	Recovery/ %	Variation
a) Hemisphere	12.35	±2.43
b) Cone	37.07	±4.76
c) Paraboloid	28.79	±2.37
d) Pyramid	20.19	±3.20
i) Spikes 6	39.13	±2.87
j) Spikes 4	31.53	±3.91
l) D Flat 5	31.68	±3.53
m) D Flat 6	26.80	±0.48
n) D Curve 5	38.05	±2.01
o) D Curve 6	28.82	±3.15

Table S7: Water collection for samples coated with a 75/25% mix (upon the spikes) of superhydrophilic/ superhydrophobic Al_2O_3 Nanoparticles (Type 3).

Item	Recovery / %	Variation
b) Cone	24.35	±4.96
c) Paraboloid	32.58	±6.28
d) Pyramid	16.67	±4.13
i) Spikes 6	34.12	±1.47
j) Spikes 4	33.40	±2.28
l) D Flat 5	45.76	±9.24
m) D Flat 6	22.29	±3.01
n) D Curve 5	46.12	±8.31
o) D Curve 6	25.77	±3.50
